# Pathogenicity of *Streptococcus iniae* causing mass mortalities of yellow catfish (*Tachysurus fulvidraco*) and its induced host immune response

**DOI:** 10.3389/fmicb.2024.1374688

**Published:** 2024-03-22

**Authors:** Hongsen Xu, Nengbin Zhu, Yiling Chen, Huamei Yue, Meiqin Zhuo, Eakapol Wangkahart, Qianrong Liang, Rui Wang

**Affiliations:** ^1^Hubei Key Laboratory of Animal Nutrition and Feed Science, School of Animal Science and Nutritional Engineering, Wuhan Polytechnic University, Wuhan, China; ^2^Key Lab of Freshwater Biodiversity Conservation Ministry of Agriculture and Rural Affairs of China, Yangtze River Fisheries Research Institute, CAFS, Wuhan, China; ^3^Laboratory of Fish Immunology and Nutrigenomics, Applied Animal and Aquatic Sciences Research Unit, Division of Fisheries, Faculty of Technology, Mahasarakham University, Mahasarakham, Thailand; ^4^Zhejiang Fisheries Technical Extension Center, and Zhejiang Fisheries Test and Aquatic Disease Prevention Center, Hangzhou, China

**Keywords:** *Tachysurus fulvidraco*, *Streptococcus iniae*, genomic characterization, pathogenicity, immune response

## Abstract

The outbreak of mass mortality occurred in *Tachysurus fulvidraco* farm in Hubei province of China. The pathogenic strain of *Streptococcus iniae* (termed 2022SI08) was isolated and identified from diseased *T. fulvidraco*, based on morphological, physiological, and biochemical characteristics, as well as *16S rRNA* gene sequence and phylogenetic analysis. Further, the whole genome of isolate *S. iniae* was sequenced and predicted to contain one single circular chromosome of 1,776,777 bp with a GC content of 37.14%. The genomic sequence analysis showed that 2022SI08 was positive for 204 virulent and 127 antibiotic resistant genes. The experimental challenge demonstrated the high pathogenicity of the retrieved isolate of *S. iniae*, with a median lethal dosage (LD_50_) 9.53 × 10^5^ CFU/g. Histopathological examination indicated that the 2022SI08 strain could induce extensive tissue cell degeneration, necrosis, hemorrhage, and inflammation in the skin, gill, fin, spleen, liver, kidney, intestine, eye, and brain. Moreover, the innate immune enzyme activities in serum such as acid phosphatase and alkaline phosphatase were increased significantly at 24 and 48 h post infection (hpi) and then decreased at 168 hpi. The transcriptional profile of immune associated gene in *T. fulvidraco* following bacterial infection was detected at each point of time, and the results revealed clear transcriptional activation of those genes, which proving their reacting and regulatory role during the response of the host against *S. iniae* infection. The results revealed that *S. iniae* was an etiological agent in the mass mortalities of *T. fulvidraco* and this research will be conducive for increasing our understanding on pathogenesis and host defensive system in *S. iniae* invasion.

## 1 Introduction

Yellow catfish (*Tachysurus fulvidraco*), belonging to family Bagridae and order Siluriformes, is widely distributed and cultured in China and other Asian countries (Liu et al., 2010). It has become an important commercial freshwater aquaculture species own to its advantages of fresh taste, rapid growth, no intermuscular bone, and high economic value (Li et al., 2022). The total production of yellow catfish has increased rapidly, with an annual output of more than 599,801 tons in 2022, which has improved about 2.04% in comparison with those produced in 2021 (Bureau of Ministry of Agriculture and Rural Affairs of China et al., 2023). Whereas, the outbreaks of infectious diseases have gradually accumulated with the continuous spread of fish farming scale and high breeding density. Bacterial diseases are the main limiting factor for sustainable development of *T. fulvidraco* culture and there have been numerous reports involving bacterial infection, such as *Edwardsiella ictaluri* (Ye et al., 2009), *Aeromonas hydrophila* (Zhou et al., 2015), *Stenotrophomonas maltophilia* (Cao et al., 2017), *Vibrio mimicus* (Fu et al., 2021), *Flavobacterium columnare* (Wang et al., 2022), and *Streptococcus iniae* (Liu et al., 2020).

*Streptococcus iniae*, a beta-haemolytic, facultative anaerobic bacteria, is a severe Gram-positive pathogen that generally distributes in the aquatic environments (Hoshina et al., 1958). This bacterium was originally isolated from the skin lesion of a freshwater dolphin in 1976 (Pier and Madin, 1976). Fish infected by *S. iniae* could cause streptococcosis associated with typical symptoms of septicemia and meningitis. To date, the infections of *S. iniae* have been found in flounder (*Paralichthys olivaceus*) (Baeck et al., 2006), red porgy (*Pagrus pagrus*, L.) (El Aamri et al., 2010), Nile tilapia (*Oreochromis niloticus*) (Legario et al., 2020), mandarin fish (*Siniperca chuatsi*) (Luo et al., 2017), golden pompano (*Trachinotus ovatus*) (Guo et al., 2018), and Adriatic sturgeon (*Acipenser naccarii*) (Colussi et al., 2022). The widespread outbreaks of *S. iniae* in aquatic animals have seriously threatened the sustainable development of aquaculture industry. Moreover, it has been shown that *S. iniae* was also a potential zoonotic agent, which lead to soft tissue infections and sepsis in humans (Lau et al., 2006).

It is well known that fish species can successfully protect themselves from bacterial infection through improving their immune response (Smith et al., 2019). Changes in enzyme activities and transcription profiles of some immune related genes can be considered as a vital indicator of immune response in *T. fulvidraco.* Therefore, detection of these genes expression levels and enzyme activity values will benefit to monitor health status and understand the immune mechanisms in aquatic animals post bacterial infection. Whereas, limited information was available about immune response of *T. fulvidraco* after *S. iniae* infection.

In this study, the *S. iniae* responsible for disease outbreaks of yellow catfish was identified, and its pathogenicity and histopathology were investigated in healthy *T. fulvidraco* by challenging the fish through intraperitoneal injection. The growing characteristic and whole genome sequence of *S. iniae* were completely investigated, and the existence of virulence genes was confirmed by PCR. Moreover, activities of alkaline phosphatase (AKP), acid phosphatase (ACP), and lysozyme (LZM) in serum, as well as expression profiles of immune-related genes at each point of time in liver and head kidney were also assessed to reveal the initiation of defense mechanism of *T. fulvidraco* after *S. iniae* infection. Results of the present research will be conducive to provide future insights for *S. iniae* characters, and increase our understanding on the pathogenesis and host defensive system in *S. iniae* invasion.

## 2 Materials and methods

### 2.1 Case history

In August 2022, an outbreak of disease coincident with considerable mortality was occurred in *T. fulvidraco*, which were cultured in four 5–6 acre earthen ponds in Zhijiang fish farm, Yichang, Hubei province, China. The diseased fish were between 150 and 300 g in body weight, and 15–22 cm in body length. Between 300 and 700 approximated fish deaths were recorded daily for 5 successive days, with mortality rate reached the peak level at around 6th day after first obvious mass mortality. Twenty moribund fish were transported timely to the laboratory in oxygen bags for diagnosis.

### 2.2 Bacteriological assay

The tissues (liver, spleen, head kidney, eye, and brain) were aseptically sampled from moribund fish and streaked directly on brain heart infusion (BHI, HopeBio, China) agar plates with inoculating loop. The agar plates were cultured at 28°C for 48 h and then the bacterial colonies were re-inoculated on BHI agar plates thrice to get the pure culture. The isolate from the above pure culture was inoculated on BHI agar supplemented with 5% defibrinated sheep blood and incubated at 28°C for 48 h to observe colony morphology and hemolytic activity. After that, the morphology of *S. iniae* from cultured agar and naturally infected fish liver were detected with a Gram Stain Kit (Solarbio, Beijing, China). Images were photographed using 100 × oil immersion objective lens on an optical microscope. The biochemical characteristics of *S. iniae* were identified using standardized API® 20 strip (BioMerieux S.A., France) according to the guidelines of the manufacturer. The *16S rRNA* gene sequencing and phylogenetic tree construction were conducted to identify the bacterial species as reported previously (Xu et al., 2022). Briefly, phylogenetic tree was constructed using the neighbor-joining algorithm in the MEGA X software package following multiple sequence alignments (using CLUSTAL W) of *16S rRNA* gene sequence. After that, the *16S rRNA* gene sequence of isolated *S. iniae* was submitted to the National Center for Biotechnology Information (NCBI) database and accession numbers was obtained. In addition, the lactate oxidase (*Lox*) gene was amplified and visualized on 1% (w/v) agarose gel containing 4S Green for visualization.

### 2.3 Whole genome sequencing of the pathogen

Strain 2022SI08 was inoculated in BHI broth at 28°C, kept shaking at 180 rpm for 10 h, and then collected by centrifugation at 10,000 *g* for 10 min. The total genomic DNA was extracted from isolated bacteria with a TIANamp bacteria DNA Kit (Tiangen, Beijing, China) as per the manufacture’s instruction and subjected to whole genome sequencing using Illumina NovaSeq 6000 sequencing platform (Shanghai Biozeron Biotechnology Co., Ltd) as reported previously (Li S. et al., 2023; Ramadan et al., 2023). Briefly, reads were assembled with SOAP *de novo* version 2 and the circular genome map was built with Circos software. The coding sequences (CDSs), transfer RNA (tRNA) and ribosomal RNA (rRNA) genes were detected using Glimmer 3.02, GeneMarkS and tRNAscan-SE, respectively. Moreover, the obtained CDSs were annotated using the Kyoto Encyclopedia of Genes and Genomes (KEGG) database, virulence related genes were analyzed by comparison with the virulence factors database (VFDB), and antimicrobial resistance genes were detected through comprehensive antibiotic resistance database (CARD).

### 2.4 Growth characteristics

The different pH value and NaCl concentration were prepared following previous study with slight modification (Li S. et al., 2023). In brief, the pH values of BHI were adjusted to 5.5, 6.0, 6.5, 7.0, 7.5, 8.0, and 8.5 with HCl (1 mol/L) or NaOH (1 mol/L) prior to sterilization. Likewise, the concentrations of NaCl in BHI were adjusted to 0.5, 1.0, 1.5, 2.0, 2.5, and 3.0%, respectively, before sterilization. The *S. iniae* isolate was cultured in BHI broth until the OD600 value reached 1.0. After that, the bacteria were introduced into prepared BHI at a ratio of 1:100 and added 200 μL of each to a 96-well plate. Growth feature of isolated 2022SI08 was monitored for 60 h by reading the OD600 value every 2 h using full-automatic microbial growth curve analyzer (Scientz MGC-200, Ningbo, China).

### 2.5 Identification of virulence biosynthesis genes

The *S. iniae* were conducted for PCR amplification to confirm the presence of genes coding virulence factors, such as C5α peptidase (*scpI*), polysaccharide deacetylase (*pdi*), SiM protein A (*simA*), capsular polysaccharide (*cpsB*, *cpsC*, and *cpsD*), CAMP factor-like (*cfi*), phosphoglucomutase (*pgm*), β-haemolysin (*tagU*, *nisF*, *YkpA*, and *ydfG*), and hyaluronic acid capsule (*hasA*). The PCR amplifications were performed in reaction volume of 25 μL mixtures containing 1 μL of DNA template (about 100 ng), 2.5 μL of 10 × Taq reaction buffer, 1.0 μL (10 pmol) of each reverse and forward primers, 2.0 μL of dNTP mix (2 mM), 0.5 μL of Taq DNA polymerase (5 U/μL), and 18.0 μL of DNase-free water. Takara thermal cycler TP600 (Takara Bio Inc. Shiga-Ken, Japan) was utilized to provide the following amplification reactions which started with a primary denaturation for 10 min at 95°C, followed by 30 cycles including a secondary denaturation for 30 s at 95°C, annealing at specified temperature (Table 1) for 30 s, extension at 72°C for 45 s, and final extension for 7 min at 72°C. The PCR products were detected by electrophoresis on 1% agarose gel stained with 4S Green. Gels were visualized and photographed using Gel Doc EZ Imager (Bio-Rad, Germany).

**Table 1:** Primers used for virulence genes in this study.

Gene	F: Primer sequence (5′–3′)	R: Primer sequence (5′–3′)	Virulence factors	Product size (bp)	GenBank ID
*scpI*	GCAACGGGTTGT CAAAAATC	GAGCAAAAGGAGTTGCTTGG	C5a peptidase	822	gene0773
*simA*	AATTCGCTCAGCAGGTCTTG	AACCATAACCGCGATAGCAC	M-like protein	994	gene0297
*pdi*	TTTCGACGACAGCATGATTG	GCTAGCAAGGCCTTCATTTG	Polysaccharide deacetylase	381	gene1804
*pgm*	TATTAGCTGCTCACGGCATC	TTAGGGTCTGCTTTGGCTTG	Phosphoglucomutase	490	AY846302
*cfi*	ATGAACTCTCAACACATTTTACG	TTAGTTAAGAGCAGCTGTTAAGG	CAMP factor	771	gene0120
*CpsB*	ATGATTGACATCCATTCCCA	CTATAAATAATCATTTTCAATCAGG	Capsular polysaccharide	732	gene0066
*CpsC*	ATGAACACAAGCGAAAACA	TTACATTTTATTTGTGTTTGGA		690	gene0067
*CpsD*	TGGTGAAGGAAAGTCAACCAC	TCTCCGTAGGAACCGTAAGC		534	gene0068
*tagU*	ATGGCACATTCCAGAAGTAA	TCATTTACTTTCCTCCATTACT		1,464	gene0065
*nisF*	ATGACTAACATCATTGAAACGA	TCATACCCCTTCCTTCTTTA	β-haemolysin	699	gene1900
*YkpA*	TTGCTTACTGTTTCTGATGTG	TTATTTCCAAAGTTCTGCAA		1,620	gene0655
*ydfG*	ATGCTTAAAAAAATTGCTCTT	TTAATCCCTATGAACCGGT		759	gene0928
*hasA*	ATGGAAAAACTGAAAAATCTAA	TTAAGTAAGAGGTTCTTCTTCCT	Hyaluronic acid capsule	1,242	gene1372

### 2.6 Antibiotic susceptibility testing

The antibiotic susceptibility of isolate 2022SI08 was determined using the disk diffusion method of Bauer (1966). In brief, 100 μL of bacterial suspension was directly swabbed onto Mueller–Hinton agar (Solarbio, Beijing, China) supplemented with 5% sheep blood, and 29 antibiotic disks (Hangzhou Tianhe Microorganism Reagent Co., Ltd., China) were applied on the streaked cultures. The zones of bacterial growth inhibition were measured after 24 h of incubation at 28°C, and antibiotic sensitivity was regarded as susceptible (S), intermediate (I), or resistant (R) according to the Clinical and Laboratory Standards Institute (CLSI) guidelines (CLSI-2016).

### 2.7 Histopathological study

The skin, gill, fin, spleen, liver, kidney, intestine, eye, and brain of diseased fish were aseptically sampled and fixed with 10 % neutral buffered paraformaldehyde. Then, the fixed tissues were dehydrated in an alcohol series (50–100%), made transparent in xylene and embedded in paraffin wax. Tissue sections were sliced transversely into 5 μm thicknesses using microtome (Leica Microsystems, Wetzlar, Germany) and stained with hematoxylin and eosin (H&E). The microphotographs were captured under a light microscope (BX51, Olympus, Japan).

### 2.8 Experimental infection and sampling

The bacterial isolate was cultured on BHI broth for 48 h at 28°C and collected by centrifugation for 5 min at 10,000 *g*. The bacteria were washed three times and bacterial suspensions were adjusted to 1.2 × 10^5^, 1.2 × 10^6^, 1.2 × 10^7^, 1.2 × 10^8^, and 1.2 × 10^9^ CFU/mL with sterile PBS, respectively. Healthy yellow catfish (*n* = 400, 20 ± 1 g) were introduced from a commercial hatchery, transported to our laboratory, maintained in 250-L aquaria at 26 ± 0.5°C, and fed with commercial pellets twice daily. Feces and uneaten feed were removed by siphon. During the experiment, the water quality parameters of the aquariums were maintained at Ammonia-N of 0.03 ± 0.01 mg/L, dissolved oxygen at 7.75 ± 0.25 mg/L, and pH at 7.00 ± 0.20, respectively.

For the pathogenicity testing, a total of 180 yellow catfish were randomly divided into six groups (30 fish per group), and then the fish in group 1–5 were intraperitoneally challenged with 100 μL of abovementioned bacterial suspensions. The fish in group 6 was injected with same value of PBS and used as the control. The number of deaths were recorded for 14 consecutive days and the median lethal dosage (LD_50_) of *S. iniae* was calculated following previous research (Mabrok et al., 2024).

To analyze the activation of immune response after bacterial infection, a total of 240 yellow catfish were divided randomly into two groups in triplicate. The fish from the experimental group were intraperitoneally challenged with 20 μL of *S. iniae* suspension at the concentration of 9.53 × 10^4^ CFU/mL, while the fish from control group were injected with an equal volume of sterile PBS. The fish before injection were anesthetized with ethyl 3-aminobenzoate methanesulfonic acid (MS222, Sigma, Beijing, China), and all protocols for experiments were permitted by the Committee of the Ethics on Animal Care and Experiments at Wuhan Polytechnic University (No. WPU202211002).

### 2.9 Determination of serum non-specific parameters

Blood samples were collected from caudal veins at 12, 24, 48, 72, 120, and 168 h post infection (hpi) and then transferred into 1.5 mL tubes immediately. The samples were incubated at room temperature for 1 h, then centrifuged at 1,200 *g* for 10 min. Serum samples were obtained from the supernatant and then applied to detect the activities of alkaline phosphatase (AKP), acid phosphatase (ACP), and lysozyme (LZM) with Nanjing Jiancheng Bioengineering Institute kits (Jiangsu, China) as per manufacture’s protocols.

### 2.10 Detection the expression levels of immune-related genes

The liver and head kidney of *T. fulvidraco* were sampled at 12, 24, 48, 72, 120, and 168 hpi, transferred to tubes filled with RNA later solution (TaKaRa, Dalian, China). The transcriptional levels of genes, encoding the cytokines (*IL-1β*, *IL-10*, *IL-6*, *TNF-α*, and *IFN-γ*), the chemokines (*IL-8*, *CCL19*), the complement factors (*C3* and *C4*), the immuno-globulins (*IgD* and *IgM*), the cellular receptors and markers (*CD3γ*, *CD8α*, *CD83*, *MHC-2α*, and *CD28*), during the infection were analyzed following the Minimum Information for Publication of Quantitative Real-Time RT-PCR experiments MIQE guidelines (Bustin et al., 2005). The list of primers used in qRT-PCR was described in Table 2. Total RNA was extracted using TRIZOL reagent and the first-strand complementary DNA (cDNA) was synthesized from 1 μg extracted RNA by using cDNA synthesis kit (TaKaRa, Dalian, China) according to the manufacturer’s instruction. The resulting cDNA was then diluted 2-fold with RNAase free water and stored at −80°C before used as template in qPCR assays.

**Table 2:** Primers sequence in the analysis of expression of immune relevant genes by qRT-PCR.

Gene	F: Primer sequence (5′–3′)	R: Primer sequence (5′–3′)	Product size (bp)	GeneBank accession No./References
*β-actin*	TTCGCTGGAGATGATGCT	CGTGCTCAATGGGGTACT	136	EU161065.2
*IL-1β*	CAGCCTACAACCCACCAAACT	CCATTCCATCGTTCTCCTTGA	177	MF770571.1
*IL-6*	CACTATCTTGCCCTGTTCCTG	AGGCGTAAATAGTCGTGTTCTG	223	XM_027176013.1
*IL-8*	TATTCCCTCCAAGTGGCTCC	GTTCTGCTTGTTGTTATGTTGACC	161	KY218792.1
*IL-10*	TCTTCTGTAGGTTCCTCCTGCTT	GTCCTGTTGAATAGTGCGGTGT	205	XM_027144360.1
*IFN-γ*	ACTTCTGCGTATGCTGTATGGTT	TGAGTGGGTCTTCTTGGAATGAT	277	XM_027151821.1
*TNF-α*	AGGTTTTGTTGGATGTGGACG	GGGAGTGCTTGATTTCTTGTGC	186	MZ245721.1
*IgM*	TTGGACTCGTGATGCCAAGG	AACTGGCCGCCTCTTTAGAC	185	JQ067604.1
*IgD*	GAAACCTCACCTCGTATC	TTGTCCTTTCTCGCTGT	127	Kong et al. (2022)
*CD8α*	GTGAGCAGCACATCTTCATTCC	GGAGGATTGGGTAGAGTTTTGTG	169	XM_027152621.1
*CD83*	GTATGGATCGGGTGACACGCACTA	GGCTCTGGGAATCCGCTAACA	231	XM_027149864.2
*C3*	GGCAGAGCAGTCCTTATGGG	CACTCACGCTCACTTCCACA	132	GU353333.1
*C4*	ATGCTCCAGCGTTTATCGTG	CCTGCTTTTGGTCGCCTTG	183	XM_027157208.2
*MHC IIα*	CTGAAGCAGGCGCTAAACAC	CTCGGCGAACTCGTATCCTC	185	KP881738.1
*CD3γ*	GAACTGAACGAATCTTCCTTGTCT	CTGCCTCCCTTCAACCTACTG	221	XM_027138707.1
*CD28*	GGAAAGACAACATCCCAGAAATC	GCCCGTGGCATCCATAGTA	254	XM_027156081.1
*CCL19*	ATCCCAAAACGCATAATCGC	CAGATGGTCTCCTTGTATTTCTTCA	185	XM_027162840.1

The qPCR was carried out in Thermofisher QuantStudio Real-Time PCR System. The reaction was performed in 20 μL volume containing 1 μL diluted cDNA template, 10 μL 2 × SYBR® SuperMix, 1 μL (10 μM) of each forward and reverse primers and 7 μL of nuclease-free water. The reaction program was 94°C for 5 min, followed by 40 cycles of 94°C for 30 s, 60°C for 15 s, and 72°C for 10 s, then melting from 72 to 95°C. *β-actin* was utilized as a reference gene for sample normalization, and relative gene expression levels were calculated using the 2^–ΔΔCt^ method.

### 2.11 Statistical analysis

All data were expressed as mean ± standard deviation (SD) and analyzed by an un-paired Student’s *t*-test using Statistical Package for the Social Sciences (SPSS) version 23.0 software for windows (IBM, Armonk, NY, United States). The statistically significant difference was set as *p* < 0.05.

## 3 Results

### 3.1 Clinical signs and gross pathology

**Figure 1:**
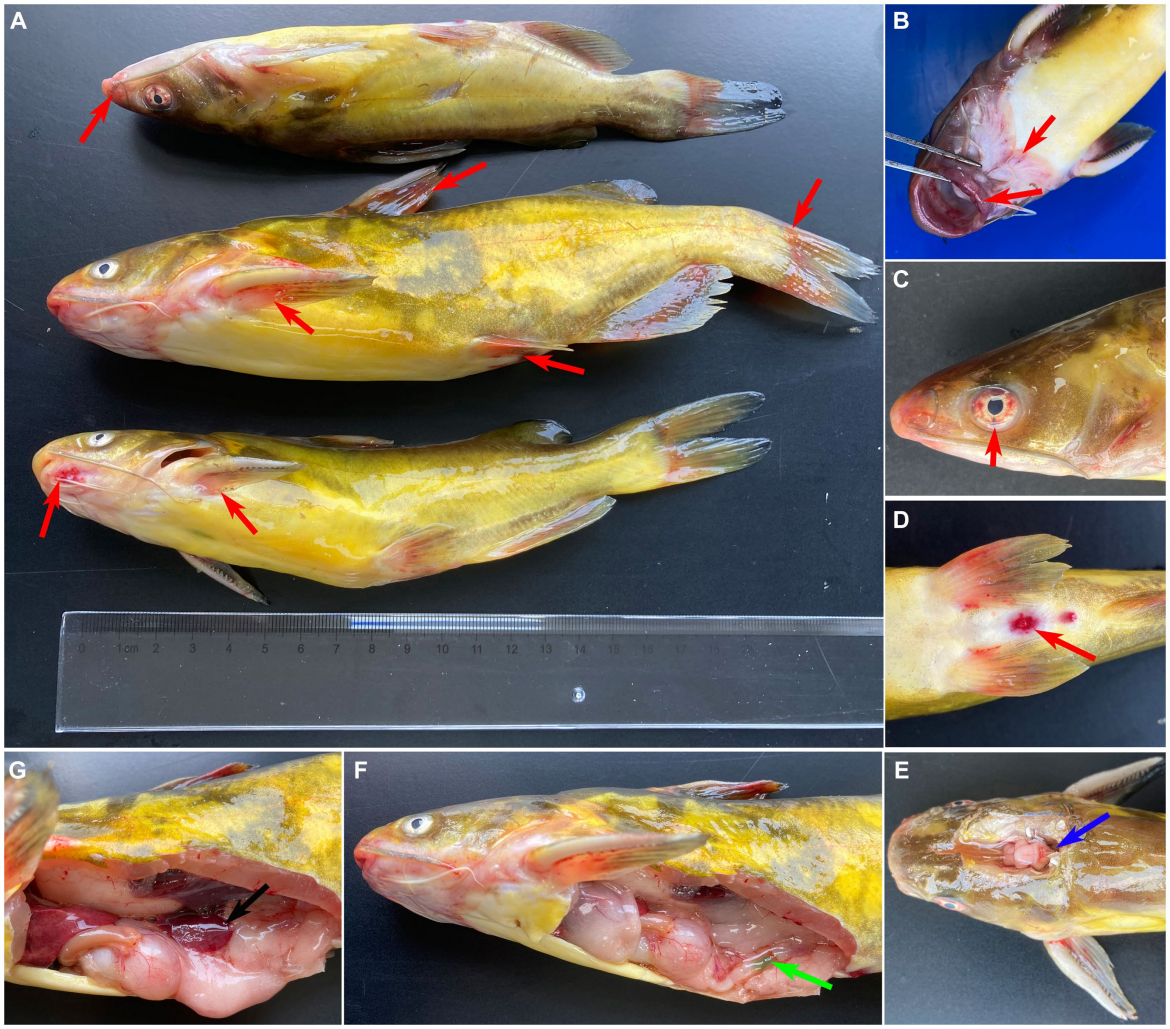
Clinical signs of naturally infected *Tachysurus fulvidraco.* **(A)** Hemorrhages are visible at pectoral, dorsal, pelvic, anal and caudal fins (red arrow). **(B)** Hemorrhage in snout, mandible, and mouth (red arrow). **(C)** Hemorrhage of eye (red arrow). **(D)** Hemorrhage of anal fin, redness and swelling of the anus (red arrow). **(E)** Cerebral oedema and hemorrhaging (blue arrow). **(F)** Green excrement in the intestine (green arrow). **(G)** The spleen is swollen (black arrow).

The diseased fish showed the clinical sign of anorexia, erratic swimming, rotating on the surface, and along with severe mortality (approximate 60%). As shown in Figure 1, the clinical sings of naturally diseased yellow catfish were associated with mass external symptom, including hemorrhages on fin (Figure 1A), mouth (Figure 1B) and eye (Figure 1C), as well as redness and swelling of the anus (Figure 1D). Moreover, some diseased fish showed cerebral oedema and hemorrhaging (Figure 1E). At necropsy, enlarged spleen (Figure 1G) and green excrement (Figure 1F) in the intestine were observed.

### 3.2 Bacteriological assay

**Figure 2:**
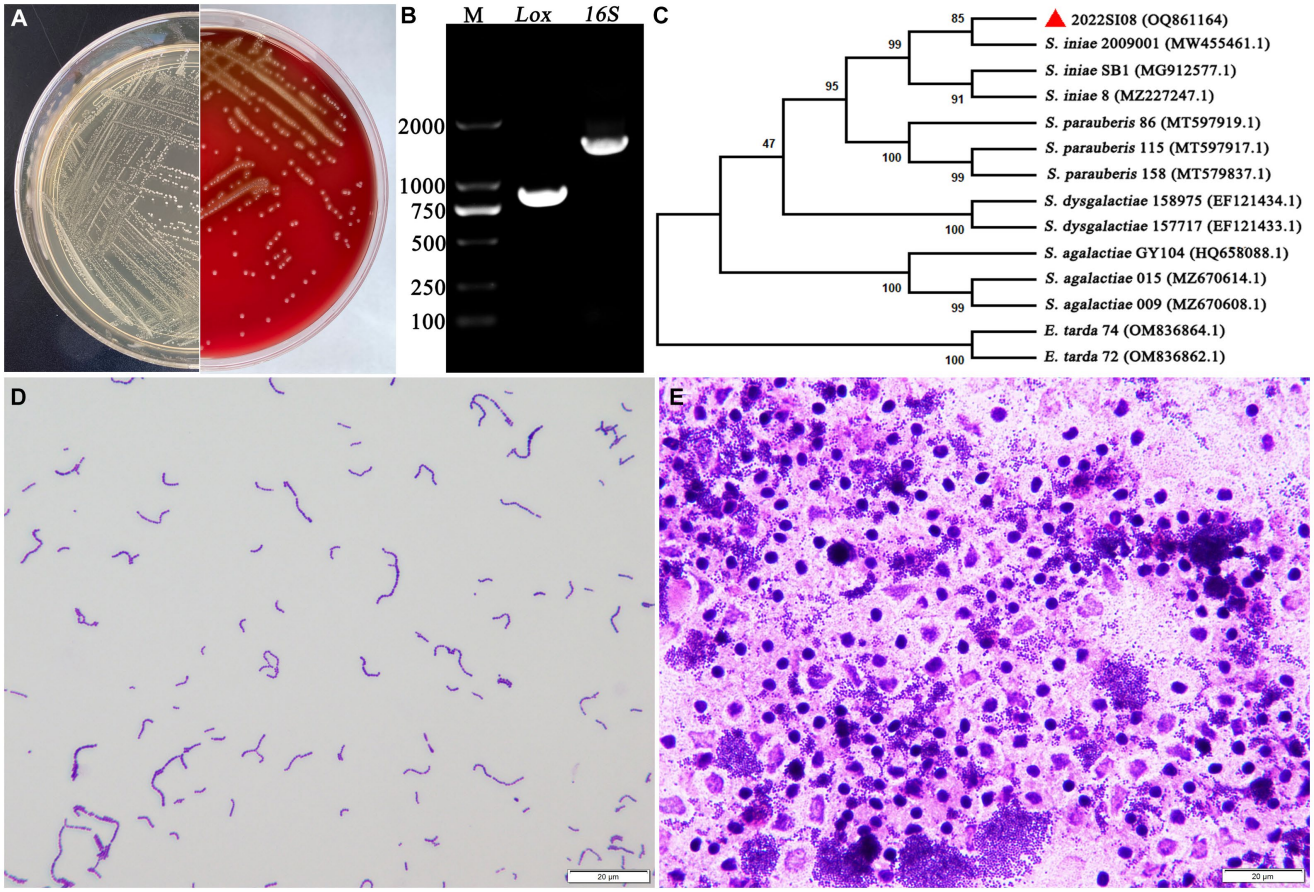
The morphological characteristics and identification of *Streptococcus iniae* strain 2022SI08. **(A)** The morphology of *S. iniae* cultured on BHI agar (left) and sheep blood agar (right) at 28°C for 24 h. The isolated 2022SI08 formed smooth, round, moist, neat-edged and milky white colonies on BHI and exhibited β-haemolysis on sheep blood agar. **(B)** Agarose gel electrophoresis showed the amplification of approximately 1,500 and 870 bp fragments of *16S rRNA* and *Lox* genes from the isolated 2022SI08, respectively. M, Trans 2 K DNA Marker; Lox, lactate oxidase gene; 16S, *16S rRNA* gene. **(C)** Phylogenetic tree analysis based on *16S rRNA* gene sequence revealed that the isolate 2022SI08 was grouped together with known species of *S. iniae*. **(D)** *Streptococcus iniae* cultured on BHI was Gram-positive and indicated in blue-purple and chain or pair-like cocci. Scale bars = 20 μm. **(E)** *Streptococcus iniae* form the kidney of natural diseased fish was Gram-positive and showed blue-purple and single-like cocci. Scale bars = 20 μm.

The isolate 2022SI08 formed smooth, round, moist, neat-edged and milky white colonies on BHI (Figure 2A, left) and produced β hemolysis on 5% sheep blood agar (Figure 2A, right). Regarding the biochemical tests, the *S. iniae* 2022SI08 showed acid producers from ribose, glycogen, mannitol, while they were failed to produce acid from lactose, l-arabinose, sorbitol, inulin, and raffinose. The strains produced pyrrolidinyl arylamidase, alkaline phosphatase, and leucine arylamidase; α-galactosidases, β-galactosidases, and acetoin were not produced. Moreover, esculin was hydrolyzed, but arginine dihydrolase and hippurate not (Table 3). The PCR assay resulted in the amplification of approximately 1,500 and 870 bp fragments of *16S rRNA* and *Lox* genes from the isolated 2022SI08, respectively (Figure 2B). The *16S rRNA* gene of *S. iniae* 2022SI08 was deposited in GenBank with an accession No. OQ861164 and displayed highest identity with the *16S rRNA* gene of *S. iniae* strain 2,009,001 (GenBank accession No. MW455461.1) after BLAST alignments. Accordingly, the phylogenetic tree revealed that the isolate 2022SI08 was grouped together with known species of *S. iniae* (Figure 2C). Moreover, *S. iniae* from either BHI medium or flesh liver of naturally infected fish showed a positive reaction with blue-purple staining and displayed chain, pair and even single-like cocci morphology, respectively (Figures 2D,E). Based on these data, the isolated 2022SI08 strain from diseased *T. fulvidraco* was identified phenotypically and molecularly as *S. iniae*.

**Table 3:** API 20 result for isolate and reference strains of *Streptococcus iniae.*

Items	*S. iniae* 2022SI08	S. iniae*
Gram stain	+	+
Cell morphology	Cocci	Cocci
Motility	−	−
Hemolytic reaction	β	β
Catalase	−	−
Oxidase	−	−
Acid from		
Ribose	+	+
Glycogen	+	+
Mannitol	+	+
Lactose	−	−
L-arabinose	−	−
Sorbitol	−	−
Inulin	−	−
Raffinos	−	−
Hydrolysis of		
Arginine dihydrolase	−	−
Esculin	+	+
Hippurate	−	−
Production of		
Pyrrolidinyl arylamidase	+	+
Alkaline phosphatase	+	+
Leucine arylamdiase	+	+
α-galactosidase	−	−
β-galactosidase	−	−
Acetoin (V–P)	−	−

“+,” positive, “–,” negative.

^*^Reference strain data compiled from Bergey’s manual (Bergey, 1994).

### 3.3 General genomic features of *Streptococcus iniae*

**Figure 3:**
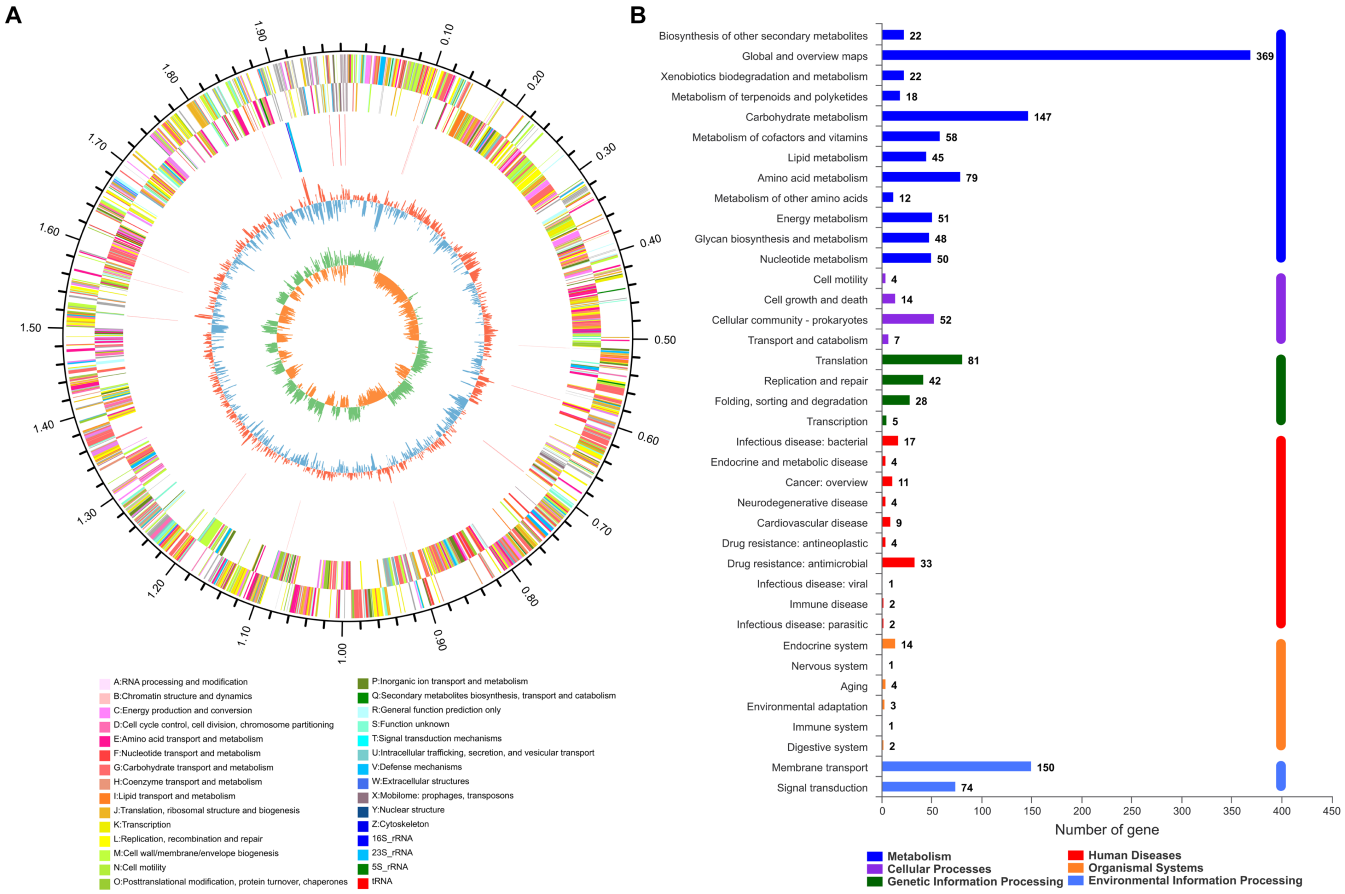
The whole genome analysis of *Streptococcus iniae* strain 2022SI08. **(A)** Circular complete genome map of isolate 2022SI08. From outside to inside, the first and fourth circles showed forward and reverse CDSs with different colors representing different COG functional classifications (right-top of map). The second and third circles showed the CDS, tRNA, and rRNA genes on positive and negative strands, respectively. The fifth and sixth circles showed the GC content plot and the GC-skew. COG, Clusters of orthologous groups; CDS, Coding sequence; tRNA, Transfer RNA; rRNA, Ribosomal RNA; and GC, Guanine-cytosine. **(B)** Statistics of functional classification of Kyoto Encyclopedia of Genes and Genomes (KEGG) protein of isolate 2022SI08.

The whole genome sequence data of isolate 2022SI08 were deposited at the NCBI BioSample database with accession No. PRJNA907543. The whole genome sequence analysis showed that the total genome of *S. iniae* 2022SI08 was 1,776,777 bp long, with an average GC content of 37.14% (Figure 3A). A total of 1,909 CDSs, 35 tRNA and three rRNA were identified in the 2022SI08 genome. Besides, 1,490 protein-coding functions were annotated using the KEGG database (Figure 3B). Among these encoded proteins, 61.80% were associated with metabolic functions, 5.17% were related to cellular processes, 10.47% were linked with genetic information processing, 1.68% were related to organismal systems, and 15.03% were associated with environmental information processing functions. Moreover, 5.84% of the genes were related to disease, of which 37 genes were associated with drug resistance and 20 genes were linked to infectious diseases.

### 3.4 Growth features of *Streptococcus iniae* 2022SI08

**Figure 4:**
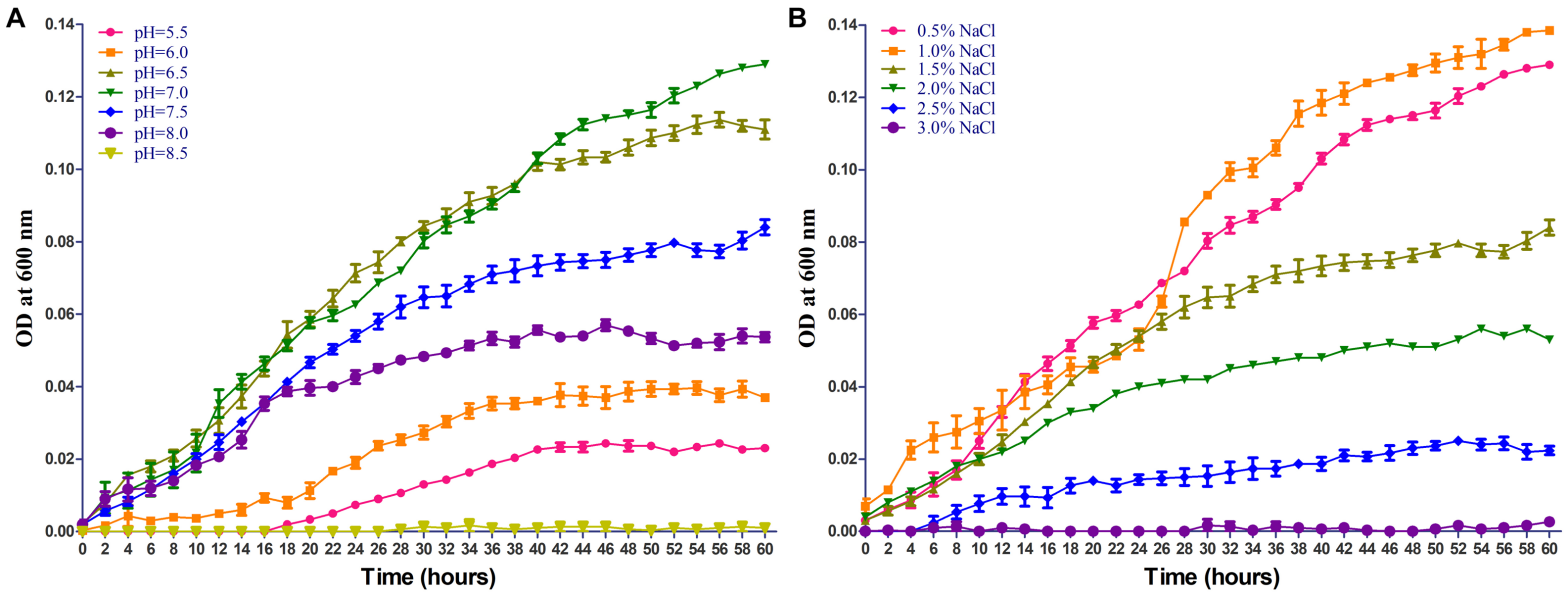
Growing characteristics of *Streptococcus iniae* 2022SI08 strain at different pH and NaCl concentration. **(A)** Growth of *S. iniae* 2022SI08 at different pH. **(B)** Growth of *S. iniae* 2022SI08 at different NaCl concentration.

The growing characteristics of 2022SI08 isolate on different pH and NaCl values were tested. As shown in Figure 4A, the *S. iniae* 2022SI08 could grow well at pH 6.5–7.5, whose optimal value was around pH 7. The latent phase of bacterial growth was somewhat prolonged at pH 6.0, and extended significantly when the pH value reached 5.5, whereas the growth was suppressed at pH 8.5. As shown in Figure 4B, the *S. iniae* 2022SI08 could grow normally at 0.5–1.5% NaCl, whose optimal value was around 1.0% NaCl. Whereas, the latent phase of bacterial growth was extended markedly at 2.0% NaCl and the growth was halted when the NaCl concentration reached 3.0%.

### 3.5 Pathogenicity analysis of *Streptococcus iniae* 2022SI08

**Figure 5:**
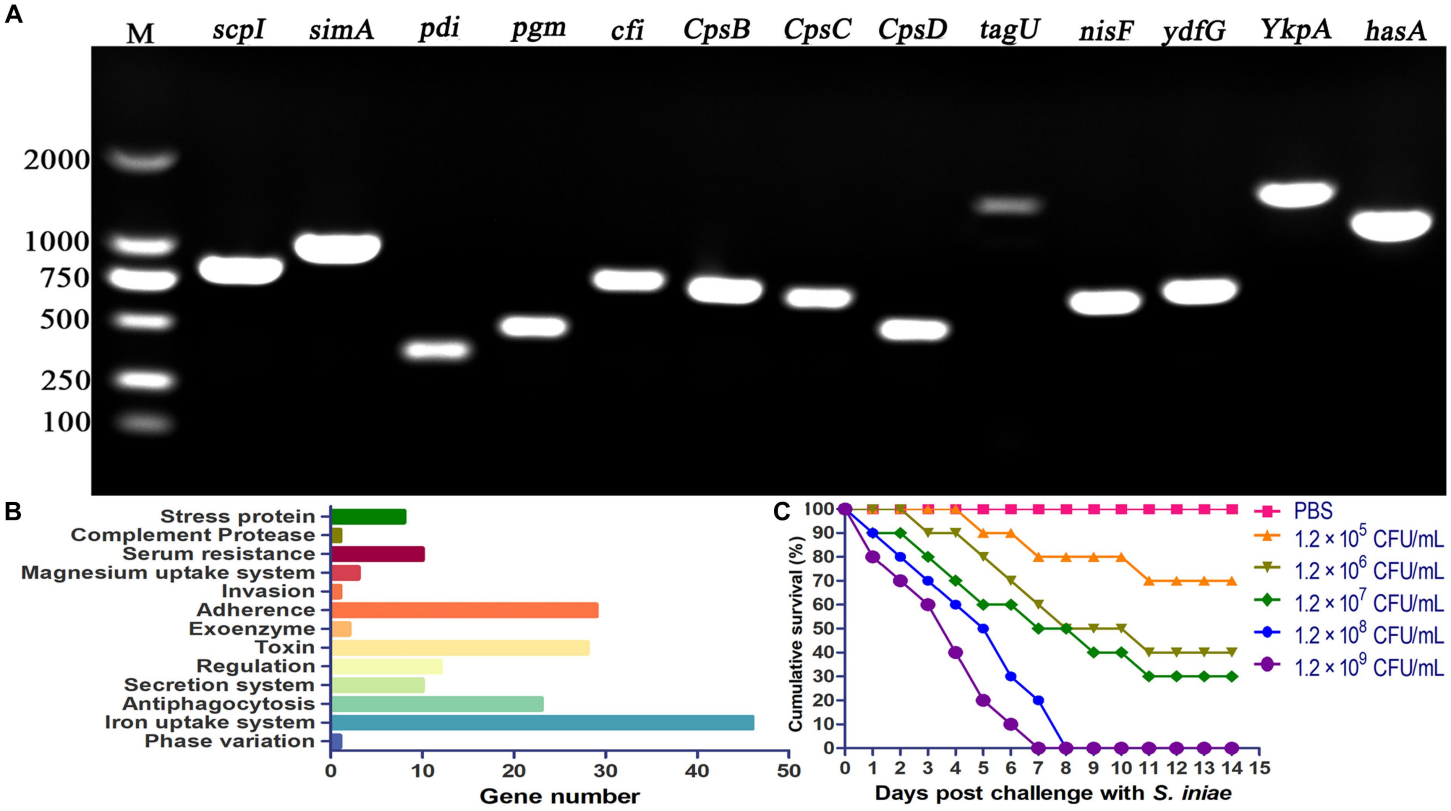
Pathogenicity of *Streptococcus iniae* 2022SI08 strain. **(A)** Agarose gel electrophoresis of the amplification products of virulent genes (*simA*, *scpI*, *pdi*, *pgm*, *sagA*, *cfi*, *cpsB*, *cpsC*, *cpsD*, *tagU*, *nisF*, *YkpA*, *ydfG*, and *hasA*). M, Trans 2 K DNA Marker. **(B)** Classification of virulent factors. **(C)** Cumulative mortalities of yellow catfish infected with different concentrations of *S. iniae*.

PCR amplification was performed to verify the presence of virulence genes in *S. iniae* 2022SI08. The results revealed that 15 virulence-related genes (*scpI*, *simA*, *pdi*, *sagA*, *pgm*, *cfi*, *cpsB*, *cpsC*, *cpsD*, *tagU*, *nisF*, *YkpA*, *ydfG*, and *hasA*) were positive for isolated *S. iniae* 2022SI08 (Figure 5A). In this study, a total of 204 virulence genes were screened from the *S. iniae* 2022SI08 after comparison by whole genome sequence (Supplementary Table S1). These virulence genes were mostly associated with processes of iron uptake, adherence, invasion, and antiphagocytosis (Figure 5B). The yellow catfish injected with 100 μL of 1.2 × 10^6^ to 1.2 × 10^9^ CFU/mL bacteria were rapidly died from the 3rd to 7th day post-injection, accompanied with the symptoms of exophthalmia and pterygiophore hemorrhage, whereas no death was observed in the fish injected with PBS during the experimental challenge. The cumulative mortality of infected fish was displayed in Figure 5C, and the LD_50_ value of *S. iniae* 2022SI08 was calculated as 9.53 × 10^5^ CFU/g fish.

### 3.6 Determination of antimicrobial resistance

The antibiotic resistance profiles of *S. iniae*, valued in terms of the inhibition zones surrounding each disk, was exhibited in Table 4. Isolate 2022SI08 was resistant (R) to kanamycin, streptomycin, amikacin, gentamycin, cefpimizole, ampicillin, rifampicin, trimethoprim, and sulfisoxazole, intermediate sensitive (I) to minocycline, carbenicillin, meropenem and imipenem, and susceptible (S) to several antibiotics including neomycin, ceftriaxone, ceftazidime, cefepime, doxycycline, tetracycline, piperacillin, oxacillin, enrofloxacin, norfloxacin, ciprofloxacin, levofloxacin, florfenicol, polymyxin B, vancomycin, and colistin.

**Table 4:** Antibiotics sensitivity of *Streptococcus iniae* isolate.

Antibiotics	Concentration/disk	Colony diameter			Mean inhibition zone diameter (mm)/Sensitivity
		R/mm	I/mm	S/mm	
Aminoglycosides					
Kanamycin	30 μg	≤13	14 ~ 17	≥18	13/R
Streptomycin	10 μg	≤11	12 ~ 14	≥15	0/R
Amikacin	30 μg	≤14	15 ~ 16	≥17	6/R
Gentamycin	10 μg	≤12	13 ~ 14	≥15	8/R
Neomycin	30 μg	≤12	13 ~ 16	≥17	19/S
Cephalosporins					
Ceftriaxone	30 μg	≤13	13 ~ 21	≥21	31/S
Cefpimizole	30 μg	≤14	15 ~ 17	≥18	0/R
Ceftazidime	30 μg	≤17	18 ~ 20	≥21	26/S
Cefepime	30 μg	≤14	15 ~ 17	≥18	20/S
Tetracyclines					
Doxycycline	30 μg	≤12	13 ~ 15	≥16	25/S
Tetracycline	30 μg	≤14	15 ~ 18	≥19	20/S
Minocycline	30 μg	≤14	15 ~ 18	≥19	17/I
Penicillin					
Ampicillin	10 μg	≤13	14 ~ 16	≥17	0/R
Piperacillin	100 μg	≤17	18 ~ 20	≥21	23/S
Carbenicillin	100 μg	≤18	19 ~ 23	≥24	21/I
Oxacillin	1 μg	≤10	11 ~ 12	≥13	20/S
Quinolones					
Enrofloxacin	5 μg	≤16	17 ~ 22	≥23	35/S
Norfloxacin	10 μg	≤12	13 ~ 16	≥17	35/S
Ciprofloxacin	5 μg	≤15	16 ~ 20	≥21	25/S
Levofloxacin	5 μg	≤12	13 ~ 16	≥17	20/S
Florfenicol	30 μg	≤12	13 ~ 17	≥18	25/S
Rifampicin	5 μg	≤16	17 ~ 19	≥20	15/R
Polymyxins					
Polymyxin B	300 μg	≤8	8 ~ 11	≥12	20/S
Vancomycin	30 μg	≤9	10 ~ 11	≥12	25/S
Colistin	10 μg	≤8	8 ~ 11	≥12	15/S
Carbapenemes					
Meropenem	10 μg	≤18	18 ~ 33	≥34	25/I
Imipenem	10 μg	≤13	14 ~ 15	≥16	15/I
Sulfonamides					
Trimethoprim	25 μg	≤14	15 ~ 17	≥18	8/R
Sulfisoxazole	30 μg	≤10	11 ~ 15	≥16	7/R

S, Susceptible; I, Intermediate; R, Resistant.

Besides, the CARD comparison results revealed that 127 antibiotic resistance genes against 25 groups of antibiotics were positive for isolate *S. iniae* 2022SI08 (Supplementary Table S2), of which coding for macrolide, tetracycline, and fluoroquinolone antibiotics occupy the largest percentage (Supplementary Figure 1).

### 3.7 Histopathology in yellow catfish infected by *Streptococcus iniae*

**Figure 6:**
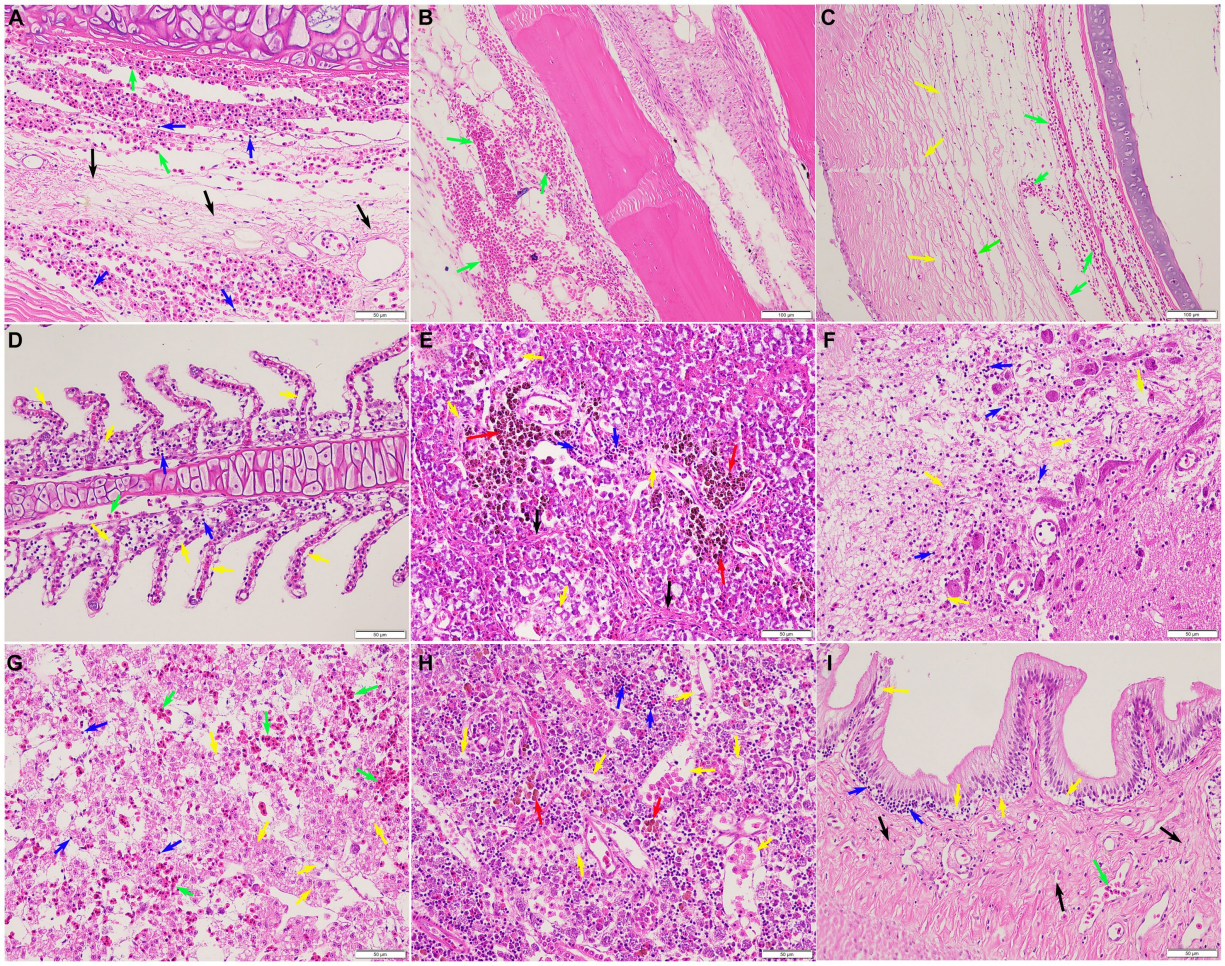
Histopathological lesions of the *Tachysurus fulvidraco* naturally infected with *Streptococcus iniae*. **(A)** Severe hemorrhage (green arrow), fibrinoid degeneration (black arrow), and inflammatory cell infiltration (blue arrow) in the skin. **(B)** Large scale of hemorrhage (green arrow) was observed in the fin. **(C)** Severe hemorrhage (green arrow), vacuolar degeneration, and necrosis (yellow arrow) in the eye. **(D)** Large scale of hemorrhage (green arrow), necrosis (yellow arrow), and inflammatory cell infiltration (blue arrow) in the gill. **(E)** Proliferation of melano-macrophage centers (red arrow), necrosis (yellow arrow), fibrinoid degeneration (black arrow), and inflammatory cell infiltration (blue arrow) in the spleen. **(F)** Meningoencephalitis showed in the brain, with necrosis (yellow arrow), oedema, and inflammatory cell infiltration (blue arrow). **(G)** Liver with focal necrosis (yellow arrow), severe hemorrhage (green arrow), and amount of inflammatory cells infiltration (blue arrow). **(H)** Degeneration and tubular necrosis (yellow arrow), inflammatory cell infiltration (blue arrow), and proliferation of melano-macrophage centers (red arrow) in the kidney. **(I)** Necrosis (yellow arrow), inflammatory cell infiltration (blue arrow), and fibrinoid degeneration (black arrow) in the intestine. Scale bars = 50 μm.

Histopathological analysis revealed that pathological changes of extensive tissue cell degeneration, necrosis, hemorrhage, and inflammation were observed in the skin, gill, fin, spleen, liver, kidney, intestine, eye, and brain from naturally infected fish. Specifically, the skin tissues of diseased fish showed severe hemorrhage and fibrinoid degeneration with inflammatory cell infiltration (Figure 6A). Large scale of hemorrhage was observed in the fin (Figure 6B). The eyes of diseased fish showed severe hemorrhage, vacuolar degeneration, and necrosis (Figure 6C). The gills of fish infected by *S. iniae* demonstrated large scale of hemorrhage, severe necrosis, and inflammatory cell infiltration (Figure 6D). Proliferation of melano-macrophage centers, necrosis, fibrinoid degeneration, and lymphocyte infiltration were observed in the spleen (Figure 6E). Severe hemorrhage, necrosis, and inflammatory cell infiltration were observed in both brain and liver (Figures 6F,G). In the kidney, degeneration and tubular necrosis, inflammatory cell infiltration, and proliferation of melano-macrophage centers were detected (Figure 6H). The original structure of cells was disappeared and vacuolated, and more inflammatory cells infiltrated in the interstitium. Necrosis, inflammatory cell infiltration and proliferation of melano-macrophage centers were observed in the intestine (Figure 6I).

### 3.8 Enzyme activities following infection

**Figure 7:**
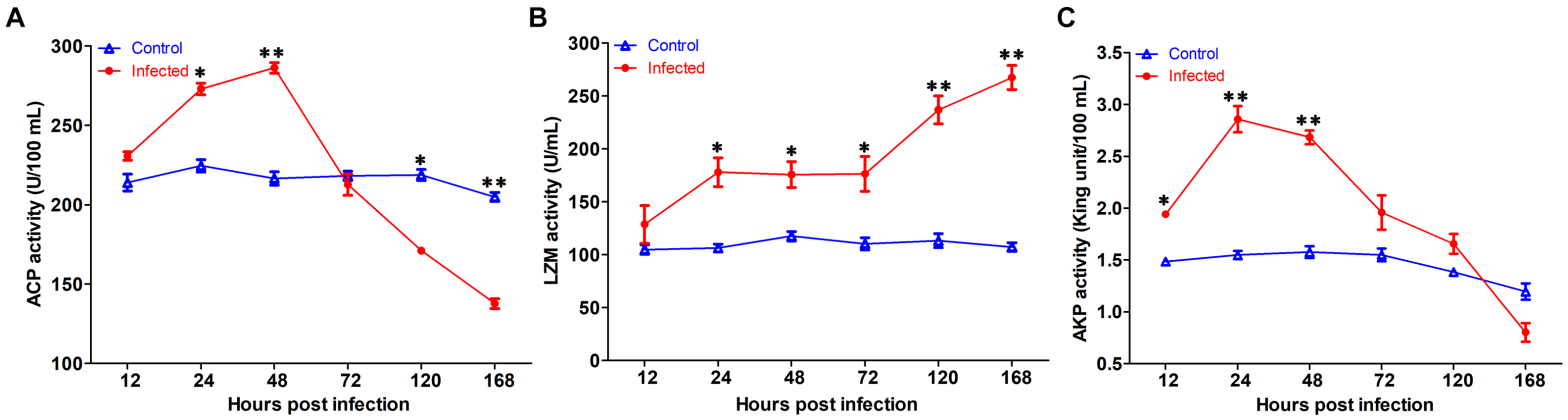
The serum non-specific immune parameters in *Tachysurus fulvidraco* infected with *Streptococcus iniae*. **(A)** Serum ACP activity. **(B)** Serum LZM activity. **(C)** Serum AKP activity. Data are representative of three independent experiments (mean ± SD). ^**^p < 0.01; ^*^0.01 ≤ *p* < 0.05.

The innate immune enzyme activities such as ACP, AKP, and LZM were measured in serum to assess the immune response of yellow catfish infected by *S. iniae* (Figure 7). The ACP activity in serum was dramatically increased at 24 hpi (*p* < 0.05), reached the summits at 72 hpi, and then decreased which was remarkably lower than that of control fish at 120 and 168 hpi (*p* < 0.05). Similarly, the activity of AKP in infected fish was upregulated significantly (*p* < 0.05) at 12 hpi, reached the maximum at 24 hpi, and then dramatically dropped (*p* < 0.05) to the value lower than that of control fish at 168 hpi. However, the LZM activity was increased obviously (*p* < 0.05) in the serum of infected fish than that from the control group.

### 3.9 Expression of immune-related genes following infection

**Figure 8:**
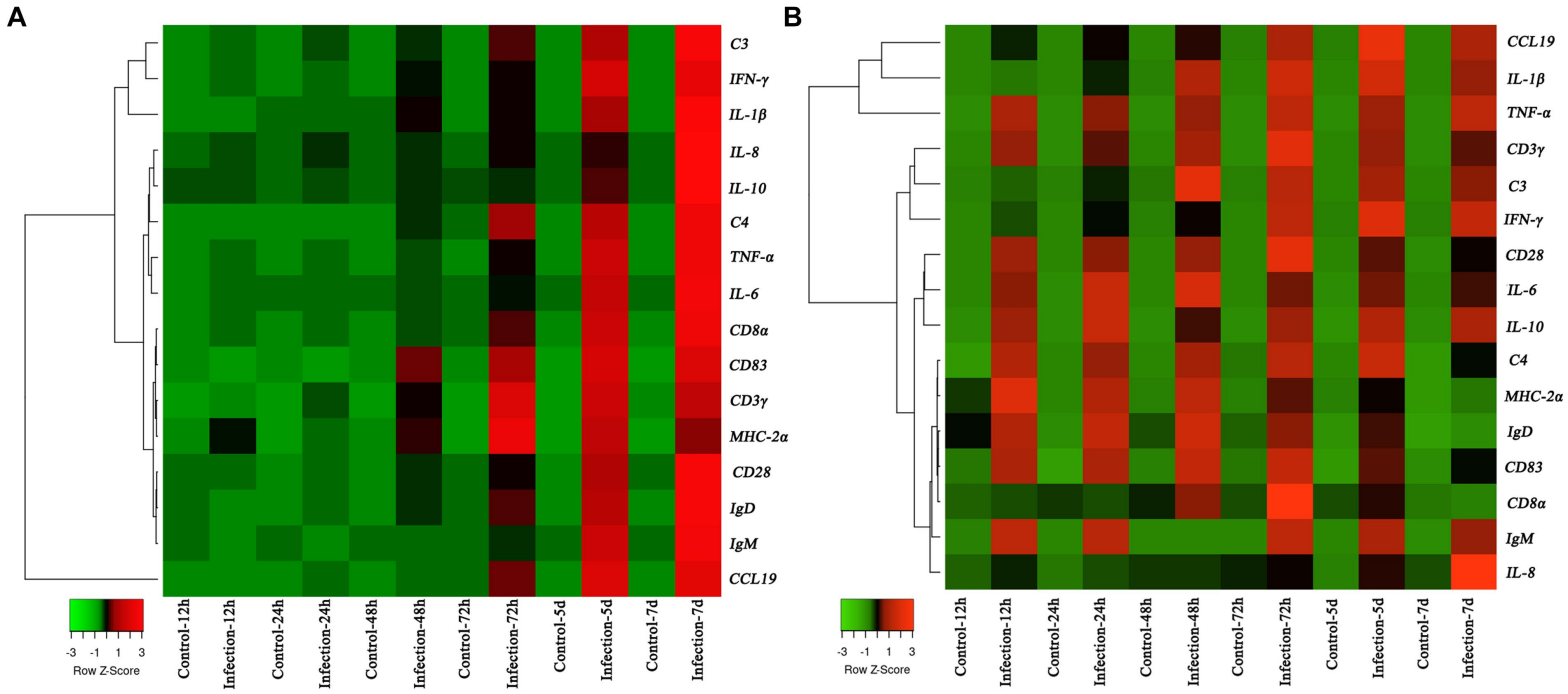
Heatmap analysis of the fold changes of immune-related genes at different time points after *Streptococcus iniae* infection measured by qRT-PCR analysis in *Tachysurus fulvidraco* liver **(A)** and head kidney **(B)**. For each gene, the mRNA level of the healthy fish without PBS injection was set as 1. Data were presented as means (*n* = 3). The color scale was shown at left bottom of the figure and presented in the color tape from low (green), mid (black), to high (red) fold changes.

The transcription profiles of immune associated genes were detected in liver and head-kidney to investigate the immune response of yellow catfish after *S. iniae* invasion (Figure 8). The transcript profiles of *CD83* and *CD3γ* genes in the liver tissue were increased obviously (*p* < 0.05) at 48 hpi with *S. iniae*, reached highest levels at 72 hpi, and then started to decrease but was still remarkably higher (*p* < 0.05) than that of control fish. The transcript levels of *C3*, *IFN-γ*, *IL-1β, IL-8*, *IL-10*, *C4*, *TNF-α*, *IL-6*, *CD8α*, *MHC-2α*, *CD28*, *IgD*, *IgM*, and *CCL19* genes were significantly enhanced (*p* < 0.05) in the liver at 72 hpi with *S. iniae*, increased gradually, and finally reached the peak level at 168 hpi. The transcript levels of *CCL19*, *IL-1β*, *IFN-γ*, and *C4* in the head kidney were found to be increased apparently (*p* < 0.05) at 48 h after *S. iniae* invasion, reached the highest level at 120 hpi, and then decreased but was still obviously greater than that of control group. Whereas, the *TNF-α*, *CD3γ*, *CD28*, *IL-6*, *IL-10*, *MHC-2α*, *IgD*, *CD83*, and *IgM* transcript levels were significantly improved (*p* < 0.05) in the head kidney of infected fish at 12 hpi in comparison with those in control fish, improved gradually, and then reached summit values at 48, 72, or 120 hpi. The transcript level of *C3* gene was increased in the head kidney from 24 to 168 hpi with *S. iniae* compared to the control group. The *CD8α* transcripts reached maximum level at 72 hpi, then decreased continuously, and finally returned to the initial level at 168 hpi. The expression of *IL-8* was obviously increased (*p* < 0.05) in the head kidney of infected fish at 72 hpi as compared with that in control fish, improved slightly, and finally reached the highest level at 168 hpi.

## 4 Discussion

*Streptococcus iniae* is an important Gram-positive streptococcal species, which could cause the outbreak of streptococcicosis, and then lead to great economic losses for global aquaculture due to its high levels of morbidity and mortality. It has been reported that the bacteria mainly cause serious diseases for various fish species cultured either from fresh water or marine water. The fish infected by *S. iniae* showed similar symptoms, including swimming erratically, rotating on the surface, septicaemia, meningitis, and panophthalmitis (Deng et al., 2017). Similarly, clinical symptom and gross lesions observed in the diseased *T. fulvidraco* were lethargy, fin rot, hemorrhages on the base of fins and skin ulcer, as well as hemorrhage in the eye. At necropsy, edematous brain and enlarged spleen were detected in diseased fish.

Whole genome sequencing has become widely used to visualize high-resolution features of bacterial pathogens (Cao et al., 2022). Recently, genomic information of many pathogenic *S. iniae* have been announced to confirm genomic diversity. For instance, the complete genome size of *S. iniae* SF1 is 2,149,844 bp with an average GC content of 36.7% and has 2,125 CDSs, 12 rRNAs, and 45 tRNAs (Zhang et al., 2014). Moreover, the complete genome of *S. iniae* 89,353 isolated from disease tilapia in Taiwan contains one single chromosome (2,098,647 bp) with 36.8% GC content and harbors 1,978 CDSs, six rRNAs, and 68 tRNAs (Gong et al., 2017). Those results are different from isolated *S. iniae* 2022SI08 in genomic features, which have a single circular chromosome (1,776,777 bp) with a GC content of 37.14% and possess 1,909 CDSs, three rRNAs, and 35 tRNAs. This phenomenon possibly due to the differences of environmental and nutritional stresses faced by microorganism (Pang et al., 2019).

The pathogenicity of bacteria is closely related to the existence of multiple virulence genes, which encode virulence factors and thereby act an important role in pathogen invasion and survival (Senderovich et al., 2012). It has been reported that *S. iniae* was positive for a variety of virulence determinants, including enzyme phosphoglucomutase, capsular polysaccharide, polysaccharide deacetylase, and the cytolysin streptolysin S, which make significant contribution to overcome host immune system, avoid phagocytic uptake, promote cellular adherence and invasion, as well as induce cell death after bacterial infection (Deng et al., 2017). M-like protein (simA) is a fibrinogen binding cell-surface protein that could protect the bacteria from oxidative attack by phagocytic cells (Baiano et al., 2008), contribute to bacterial invasion into host cells, and provide phagocytic killing resistance (Locke et al., 2008). C5α peptidase (scpI), an extracellular nuclease, contributes to hydrolyze the neutrophil chemoattractant complement factor C5α and then destroy the capacity of the infected host to fight the infection (Locke et al., 2008). Polysaccharide deacetylase (pdi) was reported to opsonize bacterial resistance to lysozyme killing and the capacity to adhere and entrance into epithelial cells (Eyngor et al., 2010). Phosphoglucomutase (pgm), a crucial factor for sugar metabolism, associated with cell wall morphology, polysaccharide capsular production, and resistance to cationic antimicrobial peptides (Buchanan et al., 2005). CAMP factor-like (cfi) has a role in binding immunoglobulin with the Fc region and thus aids in bacterial virulence (Baiano and Barnes, 2009). Capsular polysaccharide encoded by *cpsB*, *cpsC*, and *cpsD* genes exert an important role in mediating the capsule formation of *S. iniae*, and therefore protect bacterium from phagocytosis and inhibit complement C3 deposition (Miller and Neely, 2005). β-haemolysin (*tagU*, nisF, YkpA, and ydfG) play an important role in facilitating bacterial survival in macrophages and evading host immune response (Legario et al., 2020). Hyaluronic acid capsule serogroup A (hasA), virulence factor for group A streptococci, contributes to bacterial invasion by inhibiting neutrophil-mediated elimination (Hurst et al., 2022). In the present research, a total of 204 virulence-related genes were predicated by whole genome sequencing. Moreover, presence of partial virulence-associated genes, such as *scpI*, *simA*, *pdi*, *pgm*, *cfi*, *cpsB*, *cpsC*, *cpsD*, *tagU*, *nisF*, *YkpA*, *ydfG*, and *hasA*, was confirmed by PCR amplification. Similarly, previous research revealed that the *S. iniae* isolated from Adriatic sturgeon (*Acipenser naccarii*) harbored *scpI*, *simA*, *pdi*, *cfi*, *cpsD*, and *pgm* genes (Colussi et al., 2022). The pathogen can be confirmed as highly virulent when the value of LD_50_ is in the range of 1.7 × 10^4^ to 1.0 × 10^6^ CFU/g body weight (Santos et al., 1988). In this study, the LD_50_ of *S. iniae* was found to be 9.53 × 10^5^ CFU/g fish, indicating that isolated bacterium was highly virulent to yellow catfish.

According to previous reports, the disease caused by *Streptococcus* sp. results in septicaemia, meningitis, and panophthalmitis, hemorrhage in kidney, multifocal infiltrations of macrophages in spleen, granulomatous inflammation in gills, and hepatic necrosis in liver (Guo et al., 2018). Similarly, severe hemorrhage, degeneration, necrosis, and inflammatory cells infiltration were detected in the liver and head kidney. Histopathological lesions of the brain, including meningoencephalitis and oedema in the infected *T. fulvidraco* were consistent with typical pathological changes of streptococcosis infections (Chang and Plumb, 1996), strongly suggesting a preference for the brain to *S. iniae* colonization.

Multidrug resistance has escalated globally, posing a significant public health threat (Algammal et al., 2023). Recent studies have highlighted the emergence of multidrug-resistant bacterial pathogens from various sources, underscoring the imperative for judicious antibiotic use (Algammal et al., 2022a; Raharjo et al., 2023). Additionally, routine utilization of antimicrobial susceptibility testing is essential to identify suitable antibiotics and screen for emerging multidrug-resistant strains (Algammal et al., 2022b). At present, streptococcosis was mainly treated by drugs, and based on our study the *S. iniae* 2022SI08 isolate was susceptible to antibiograms, including cephalosporins, tetracyclines, quinolones, and polymyxins, which was similar to previous investigations on streptococcus (Agnew and Barnes, 2007), and the susceptibility pattern provided accurate suggestions for antibiotic utilization in disease outbreaks. The antibiotic resistance related genes could be horizontally transferred among strains through plasmids conjugation, phages transduction, and extracellular DNA transformation (Li S. et al., 2023). In the present research, the *S. iniae* 2022SI08 consisted of 127 antibiotics resistance genes screened by whole gene sequence, highlighting the risks related to the potential transmission of genes involving in resistance to antibiotics between *S. iniae* and other bacteria. Therefore, it is necessary to develop alternative strategies, such as application of proper vaccines (Sun et al., 2012) and dietary immunostimulants (Tahmasebi-Kohyani et al., 2011), to control the occurrence of streptococcosis effectively.

The innate immune system, first line of fish defense system, plays an important role when bacteria invade the body (Magnadóttir, 2006). ACP is an enzyme localized within lysosomes that plays a vital role for the intracellular digestion of phagocytized antigens and has been widely applied to reflect the degree of macrophage activation in vertebrates (Li R. et al., 2023). AKP, an extracellular enzyme, exerts an important role in hydrolyzing phosphoester bonds in various organic compounds, like proteins, carbohydrates, and lipids (Hassanatabar et al., 2013). Our study showed that both ACP and AKP activities were significantly increased at 24 and 48 hpi, and thereafter decreased, as compared with uninfected fish. Likewise, the activity of ACP in Nile tilapia was first increased at 6, 12, and 24 hpi with *Aeromonas schubertii*, and then decreased (Ren et al., 2020). LZM is a cationic enzyme found in lysosomes that acts as non-specific innate immunity molecules to induce bacteriolysis and prevent bacteria proliferation, and thus is regarded as a protective factor to defend the host against bacterial invasion, particularly those infected by Gram-positive bacterial pathogen (Khalil et al., 2023). In this study, the LZM activity was significantly improved following *S. iniae* infection, which revealed that the fish activate the innate immune system post infection. Whereas, the LZM activity in snout bream (*Megalobrama amblycephala*) was significantly reduced at 1st, 2nd, and 3rd week after *A. hydrophila* infection (Zhang et al., 2022). The differences were perhaps due to the species and infection time.

Presently, limited information was available on the molecular immunomodulation mechanism in yellow catfish after *S. iniae* infection. In the present research, we sought to evaluate the expression levels of immune-related genes in the liver and head kidney of yellow catfish by qRT-PCR. Cytokines exert essential roles in modulating inflammation and immunity, and therefore their mRNA expression levels have been employed to measure immune responses (Secombes et al., 2011). Especially, pro-inflammatory cytokines, such as *IL-1β*, *TNF-α*, *IL-8*, *IFN-γ*, and *IL-6* are frequently applied as immune-regulatory genes in fish species (Raida et al., 2011). *IL-10* is an anti-inflammatory cytokine that exerts a vital role in dampening inflammation to regulate inflammation balance (Zou and Secombes, 2016). Our finding indicated that the transcription levels of *IL-1β*, *IL-10*, *IL-6*, *TNF-α*, and *IFN-γ* were notably increased post *S. iniae* challenge, which was consistent with the expression profile in European seabass (*Dicentrarchus labrax*) infected with *S. iniae* (El Aamri et al., 2015). The complement has been viewed as a part of the innate immune system which can help to fight microbial intruders, and can quickly tag and remove them directly (Ricklin et al., 2010). Previous studies have showed that the C3 and C4 in the liver were induced significantly at transcription level after *E. ictaluri* invasion (Zhou et al., 2021). In this study, the expression levels of *C3* and *C4* genes in the head kidney and liver were upregulated distinctly and then downregulated post *S. iniae* infection. After infection, the professional antigen presenting cells will engulf the invasion bacteria, process those antigens and then present the resulting peptides in association with MHC class to T cells (Secombes and Wang, 2012), indicating the expression of *CD3γ*, *CD8α*, *CD83*, *MHC-2α*, and *CD28* genes have temporal differences after bacterial infection. In this research, the peak expression level of *CD83* and *MHC-2α* genes appeared earlier than *CD3γ*, *CD8α*, and *CD28* genes. Chemokines played an important role in regulating cell migration and immune response, and have been viewed as a link between innate and adaptive immunity (Xu and Liu, 2023). As the systemic immunoglobulin, IgM and IgD mediate the adaptive immune response by antibody dependent cell-mediated cytotoxicity, opsonisation, and complement activation (Qian et al., 2023). In this research, significantly expression of chemokines and immunoglobulin were observed, indicating the initiation of immune response after bacterial infection.

In conclusion, a highly pathogenic *S. iniae* 2022SI08 responsible for disease outbreaks of yellow catfish was isolated and identified. The complete genome of the 2022SI08 was large and harbored a total of 204 virulence genes. The early stage of *S. iniae* infection upregulated the activation of ACP, AKP, and LZM enzyme activities in serum and expression of immune related genes in yellow catfish. Our results will help to better reveal the pathogenic mechanisms of *S. iniae* and increase our understanding on the pathogenesis and host defensive system in *S. iniae* invasion.

## Data availability statement

The datasets presented in this study can be found in online repositories. The names of the repository/repositories and accession number(s) can be found at: https://www.ncbi.nlm.nih.gov/genbank/, No. OQ861164.

## Ethics statement

The animal studies were approved by Committee of the Ethics on Animal Care and Experiments at Wuhan Polytechnic University. The studies were conducted in accordance with the local legislation and institutional requirements. Written informed consent was obtained from the owners for the participation of their animals in this study.

## Author contributions

HX: Conceptualization, Data curation, Funding acquisition, Investigation, Methodology, Software, Supervision, Visualization, Writing – original draft, Writing – review & editing. NZ: Investigation, Methodology, Software, Visualization, Writing – original draft. YC: Data curation, Methodology, Resources, Writing – original draft. HY: Data curation, Resources, Writing – original draft. MZ: Data curation, Funding acquisition, Writing – original draft. EW: Resources, Writing – original draft. QL: Validation, Writing – original draft. RW: Validation, Writing – original draft.
