# Supplementary material for: Pathogenicity of Streptococcus iniae causing mass mortalities of yellow catfish (Tachysurus fulvidraco) and its induced host immune response
Source: Front Microbiol. 2024 Mar 22;15:1374688. doi: 10.3389/fmicb.2024.1374688 (PMC10995319; doi:10.3389/fmicb.2024.1374688)
Supplement: Supplementary file 1 [file Table_1.DOCX]

**Table S1.** Potential virulence factor profiles of the *S. iniae* 2022SI08 strain predicted using the virulence factor database (VFDB) Classification.

| **Class** | **Virulent factors** | **Gene** | **Gene ID** | **Position** | **Product** | **Description** | **Identity(%)** |
| --- | --- | --- | --- | --- | --- | --- | --- |
| Adherence | FBPs | - | gene0013 | 10591-12258 | fibronectin-binding domain-containing protein | Binds to fibronectin via homologous repeat region. This domain promotes fibronectin-mediated collagen recruitment, which leads to matrix deposition on and between streptococcal cells to induce the formation of large bacterial aggregates. Furthermore, collagen-recruiting streptococci were able to colonize collagen fibres and were protected from adhering to human PMN cells in the presence of opsonizing antibodies;PrtF1 also mediate internalization via integrins, fibronectin acts as an adapter molecule linking bacteria to the cell integrins. | 79.9 |
|  | Lmb | znuA | gene0359  gene1907 | 528-2084  54-803 | zinc ABC transporter substrate-binding protein AdcA  laminin binding protein | 34 kDa lipoprotein mediates the attachment to human laminin, a major component of the basement membrane, which may be essential for the bacterial colonization; also important for the invasion of human brain endothelial cells | 33.1 |
|  |  |  |  |  |  |  | 77.1 |
|  | C5a peptidase | scpI | gene0773 | 15709-19077 | S8 family serine peptidase | Cleaves C5a, the major neutrophil chemoattractant produced by activation of the complement cascade, causing an impaired recruitment to sites of infection; may function as an invasin and Fibronectin binding proteins. | 37.4 |
|  |  | - | gene1009 | 21665-26560 | S8 family serine peptidase |  | 30.3 |
|  | CBPs | - | gene1132 | 16405-17541 | SH3 domain-containing protein | CbpD, CbpE, CbpG, LytB and LytC play a role in adhesion and colonization of the nasopharynx | 41.2 |
|  | Clumping factor | - | gene1439 | 11647-13545 | - | ClfA and ClfB bind to different sites in fibrinogen. ClfA binds to the γ-chain whereas ClfB binds to the α-chain; ClfA through its fibrinogen-binding function is a mediator of S. aureus-induced platelet aggregation | 52.4 |
|  |  | - | gene1855 | 3-3743 | - |  | 42.2 |
|  | PavB | - | gene1656 | 5528-6598 | fibronectin-binding SSURE repeat-containing protein | A surface-exposed adhesion, which contributes to pneumococcal colonization and infections of the respiratory airways | 48.4 |
|  | Immune evasin | pdi | gene1804 | 5973-6893 | polysaccharide deacetylase family protein | - | 99.8 |
| Antiphagocytosis;Serum resistance | Capsule | tagU | gene0065 | 68412-69875 | LCP family protein | Inhibits the binding of the activated complement factor C3b to the surface of S. agalactiae, preventing the activation of the alternative complement pathway and inhibits complement-mediated opsonophagocytosis | 55.9 |
|  |  | CpsB | gene0066 | 69872-70603 | Tyrosine-protein phosphatase CpsB |  | 67.1 |
|  |  | CpsC | gene0067 | 70612-71301 | Capsular polysaccharide biosynthesis protein CpsC |  | 51.3 |
|  |  | CpsD | gene0068 | 71312-72031 | tyrosine-protein kinase |  | 60.7 |
|  |  | GlyA | gene1434  gene1437 | 3744-4619  7368-8249 | Glycosyltransferase GlyA |  | 33.8 |
|  |  |  |  |  |  |  | 37.6 |
|  | M protein | simA | gene0297 | 10091-11656 | LPXTG cell wall anchor domain-containing protein | Binds to complement control factors(Factor H , factor H-like protein(FHL-1), C4b-binding protein(C4BP)) and host proteins (fibrinogen) to prevent activation of the alternate complement pathway and impede phagocytosis; M protein seems to be important in adherence to Hep-2 cells in tissue culture and mediates adherence to skin keratinocytes via the attachment of the C repeat region to keratinocyte membrane cofactor CD46; involve in the internalization process (zipper like mechanism); M proteins have been suggested to play a role in the generation of an inflammatory response by binding of fibrinogen, kininogen, or plasminogen | 27.9 |
|  | Hyaluronic acid capsule | galU | gene1099 | 7078-7980 | UTP--glucose-1-phosphate uridylyltransferase GalU | GAS capsular hyaluronate is chemically quite similar to that found in human connective tissue. Thus, the capsule not only prevents phagocytosis by the usual route of discouraging C3b binding but makes the bacteria look like 'self' to the immune system; binds to CD44 to induce marked cytoskeletal rearrangements manifested by membrane ruffling and disruption of intercellular junctions, thus promotes tissue penetration by GAS through a paracellular route. Transduction of the signal involved Rac1 and the cytoskeleton linker protein ezrin, as well as tyrosine phosphorylation of cellular proteins | 90.2 |
|  |  | ugd | gene1102 | 9677-10882 | nucleotide sugar dehydrogenase |  | 67.1 |
|  |  | hasA | gene1103 | 10909-12174 | glycosyltransferase family 2 protein |  | 68.4 |
| Complement Protease | Neuraminidase | SiaA | gene1502 | 8217-10925 | exo-alpha-sialidase | Cause significant damage to host cell glycans, change the glycosylation patterns of the host and probably exposes more of the host cell surface, which may reveal surface receptors for possible interaction with the bacteria, contributing to increased adhesion | 48.9 |
| Exoenzyme | DNase | endA | gene0183 | 46968-47738 | streptodornase A type SdzA | Digesting DNA released from dead cells the enzyme reduces the viscosity of pus and allows the organism greater motility | 76.6 |
|  | Hyaluronate lyase | hysA | gene1372 | 12010-15513 | polysaccharide lyase 8 family protein | Facilitates spread of bacteria by breaking down the hyalurone polymers present in the extracellular matrices of the host; the GBS hyaluronate lyase also has limited specificity for achondroitin sulphate and cleaves the chain at unsulphated sites. This action may facilitate deep tissue penetration during infection | 52.1 |
| Toxin | CAMP factor | cfi | gene0120 | 124517-125287 | CAMP factor family pore-forming toxin | Forms pores in cell membrane by oligomerization; binds to the Fc fragments of human IgG and IgM | 60.9 |
|  | β-hemolysin | EcsA | gene0744 | 35259-35993 | ABC transporter ATP-binding protein | - | 33.9 |
|  |  | YhaQ | gene0850 | 8572-9465 |  |  | 29.8 |
|  |  | rbsA | gene1244 | 17882-19360 |  |  | 31.7 |
|  |  | ThiQ | gene0016 | 14473-15231 |  |  | 32.8 |
|  |  | livG | gene1345 | 9262-10026 |  |  | 29.6 |
|  |  | lnrL | gene1585 | 8785-9708 |  |  | 31.2 |
|  |  | cylA | gene1641 | 1838-2698 |  |  | 34.3 |
|  |  | evrA | gene1885 | 584-1576 | ATP-binding cassette domain-containing protein |  | 31.7 |
|  |  | YkpA | gene0655 | 48847-50466 |  |  | 24.8 |
|  |  | nisF | gene1900 | 299-997 | lantibiotic protection ABC transporter ATP-binding subunit |  | 35.7 |
|  |  | ydfG | gene0928 | 9711-10469 | SDR family NAD(P)-dependent oxidoreductase |  | 29.8 |
|  | SLS | sagA | gene1579 | 3656-3820 | streptolysin S family TOMM toxin | SLS lyses a wide variety of eukaryotic cells, including myocardial cells, kidney cells, platelets, lymphocytes, and neutrophils | 76.5 |
| Others | PsaA | mtsA | gene0991 | 5614-6543 | metal ABC transporter substrate-binding protein | Key function is the transport of Mn2+ and Zn2+ into the cytoplasm of the bacteria; psaA mutants show marked impact on the capacity to colonize and increased susceptibility to oxidative damage | 72.1 |
