# Supplementary material for: Pathogenicity of Streptococcus iniae causing mass mortalities of yellow catfish (Tachysurus fulvidraco) and its induced host immune response
Source: Front Microbiol. 2024 Mar 22;15:1374688. doi: 10.3389/fmicb.2024.1374688 (PMC10995319; doi:10.3389/fmicb.2024.1374688)
Supplement: Supplementary file 2 [file Table_2.DOCX]

**Table S2.** The predicted antimicrobial resistance-associated genes in the genome of isolate *S. iniae* 2022SI08.

| Drug Class | Gene ID | ARO Name | ARO Description | Identity(%) |
| --- | --- | --- | --- | --- |
| acridine dye;fluoroquinolone antibiotic | gene0848 | cdeA | Clostridioides difficile and Escherichia coli multidrug efflux transporter with antiporter function. Confers resistance to fluoroquinolones in E. coli and acriflavin in Clostridioides difficile. | 26.3 |
|  | gene1045 | arlR | ArlR is a response regulator that binds to the norA promoter to activate expression. ArlR must first be phosphorylated by ArlS. | 54.2 |
|  | gene1659 | arlR |  | 33 |
|  | gene0785 | arlR |  | 39.2 |
|  | gene1046 | arlS | ArlS is a protein histidine kinase that phosphorylates ArlR, a promoter for norA expression. | 32.8 |
|  | gene1189 | arlS |  | 33 |
|  | gene1381 | arlS |  | 31.3 |
|  | gene1660 | arlS |  | 26.4 |
|  | gene0784 | arlS |  | 36.7 |
| aminocoumarin antibiotic | gene0093 | parY | point mutation on the Streptomyces rishiriensis parY resulting in aminocoumarin resistance | 40.7 |
|  | gene1511 | parY |  | 43.6 |
|  | gene0339 | novA | A type III ABC transporter, identified on the novobiocin biosynthetic gene cluster, involved in the transport and resistance of novobiocin. | 27.1 |
| carbapenem;cephalosporin;cephamycin;monobactam;penam;penem;phenicol antibiotic | gene1545 | golS | GolS is a regulator activated by the presence of golD, and promotes the expression of the MdsABC efflux pump. | 37.7 |
| carbapenem;cephalosporin;cephamycin;penam | gene0064 | NmcR | NmcR is a homolog of the LysR regulator found in Enterobacter cloacae that contribute to the regulation of NmcA beta-lactamase | 27.3 |
| cephalosporin;penam;peptide antibiotic | gene0990 | abcA | AbcA is a multidrug resistant ABC transporter that confers resistance to methicillin, daptomycin, cefotaxime, and moenomycin. | 28.6 |
| diaminopyrimidine antibiotic | gene0711 | dfrE | dfrE is a chromosome-encoded dihydrofolate reductase found in Enterococcus faecalis | 41.8 |
| fluoroquinolone antibiotic | gene0431 | patA | PatA is an ABC transporter of Streptococcus pneumoniae that interacts with PatB to confer fluoroquinolone resistance. | 29.2 |
|  | gene0491 | patA |  | 32.9 |
|  | gene0788 | patA |  | 29.4 |
|  | gene1096 | patA |  | 66.8 |
|  | gene1095 | patB | PatB is an ABC transporter of Streptococcus pneumoniae that interacts with PatA to confer fluoroquinolone resistance.. | 67.6 |
| fluoroquinolone antibiotic;macrolide antibiotic;rifamycin antibiotic | gene0501 | efrA | efrA is a part of the EfrAB efflux pump, and both efrA and efrB are necessary to confer drug resistance. | 32.2 |
|  | gene0567 | efrA |  | 30.2 |
|  | gene0696 | efrA |  | 54.5 |
|  | gene0697 | efrB | efrB is a part of the EfrAB efflux pump, and both efrA and efrB are necessary to confer multidrug resistance. | 55.6 |
|  | gene0500 | efrB |  | 41.9 |
| fosfomycin | gene0271 | murA | Chlamydia murA confers intrinsic resistance to fosfomycin. The presence of an aspartic acid residue in place of the critical cysteine at position 119 that enables fosfomycin binding is believed to be responsible for this intrinsic resistance. | 34.1 |
| glycopeptide antibiotic | gene0143 | vanXYG | vanXYG is a vanXY variant found in the vanG gene cluster | 31.6 |
|  | gene0162 | vanYF | vanYF is a vanY variant found in the vanF gene cluster | 30.5 |
|  | gene0197 | vanTG | vanTG is a vanT variant found in the vanG gene cluster | 32.6 |
|  | gene0441 | vanHO | vanHO is a vanH variant in the vanO gene cluster | 29.9 |
|  | gene0544 | vanRA | vanRA, also known as vanR, is a vanR variant found in the vanA gene cluster | 38.5 |
|  | gene0545 | vanSL | vanSL is a vanS variant found in the vanL gene cluster | 24.3 |
|  | gene0597 | vanSG | vanSG is a vanS variant found in the vanG gene cluster | 27.5 |
|  | gene0598 | vanRF | vanRF is a vanR variant found in the vanF gene cluster | 39.9 |
|  | gene1376 | vanRE | vanRE is a vanR variant found in the vanE gene cluster | 28.6 |
|  | gene1455 | vanHB | vanHB is a vanH variant in the vanB gene cluster | 32.9 |
|  | gene1559 | vanE | VanE is a D-Ala-D-Ala ligase homolog that can synthesize D-Ala-D-Ser, an alternative substrate for peptidoglycan synthesis that reduces vancomycin binding affinity in Enterococcus faecalis | 35.1 |
| lincosamide antibiotic;macrolide antibiotic;oxazolidinone antibiotic;phenicol antibiotic;pleuromutilin antibiotic;streptogramin antibiotic;tetracycline antibiotic | gene0925 | lmrD | lmrD is a chromosomally-encoded efflux pump that confers resistance to lincosamides in Streptomyces lincolnensis and Lactococcus lactis. It can dimerize with lmrC | 29.8 |
|  | gene0211 | lmrC | lmrC is an ABC-F subfamily protein that confers resistance to lincosamides in Streptomyces lincolnensis and Lactococcus lactis. It can dimerize with lmrD | 36 |
|  | gene0507 | salA | salA is an ABC-F subfamily protein gene isolated from the chromosome of Staphylococcus sciuri conferring resistance to lincosamides and streptogramins. | 28.3 |
|  | gene0655 | optrA | OptrA is a member of the ABC-F protein subfamily that confers resistance to oxazolidinones. The gene encoding the protein was originally isolated from a plasmid in Enterococcus faecalis and Enterococcus faecium. | 29.2 |
|  | gene0761 | optrA |  | 34.8 |
|  | gene1091 | optrA |  | 26.1 |
|  | gene0020 | optrA |  | 34 |
|  | gene1373 | vmlR | vmlR is an ABC-F ATPase ribosomal protection protein identified in Bacillus subtilus. Shown to confer resistance to lincomycin and streptogramin A virginiamycin. Described by Crowe-McAuliffe et al. 2018. | 30.9 |
|  | gene0956 | erm(45) | Erm45 is an rRNA methylase that confers resistances to macrolide, lincosamide, and streptogramin B. | 26.5 |
|  | gene0890 | lmrP | lmrP is a proton motive force-dependent drug transporter that is part of the MFS efflux pump family. | 72.3 |
|  | gene1239 | cfrA | CfrA is a chloramphenicol-florfenicol resistance gene and methyltransferase enzyme. Methylation of position 8 of A2503 in 23S rRNA confers resistance to chloramphenicol antibiotics first identified by Schwarz 2000 as cfr from Staphylococcus sciuri. Additional Oxazolidinone resistance mediated by the cfr gene in a human isolated was first reported from Colombia in linezolid- and methicillin-resistant Staphylococcus aureus (PMID: 10952608). Described by Arias et al. 2008. Chandela T. et al. (PMID: 28663118) grouped Cfr with ClbA in the Cfr Group. | 37.2 |
| macrolide antibiotic | gene0016 | macB | MacB is an ATP-binding cassette (ABC) transporter that exports macrolides with 14- or 15- membered lactones. It forms an antibiotic efflux complex with MacA and TolC. macB corresponds to 1 locus in Pseudomonas aeruginosa PAO1 and 1 locus in Pseudomonas aeruginosa LESB58. | 27.8 |
|  | gene0133 | macB |  | 42.1 |
|  | gene0191 | macB |  | 29.4 |
|  | gene1658 | macB |  | 35.9 |
|  | gene0263 | macB |  | 34.1 |
|  | gene0307 | macB |  | 29.7 |
|  | gene0370 | macB |  | 34.8 |
|  | gene0508 | macB |  | 38.7 |
|  | gene0644 | macB |  | 33.6 |
|  | gene0670 | macB |  | 48.7 |
|  | gene0671 | macB |  | 32.8 |
|  | gene0754 | macB |  | 34.7 |
|  | gene0982 | macB |  | 27.8 |
|  | gene1035 | macB |  | 35.2 |
|  | gene1170 | macB |  | 36.7 |
|  | gene1758 | macB |  | 29.1 |
|  | gene1766 | macB |  | 32.2 |
|  | gene1787 | macB |  | 32.6 |
|  | gene1796 | macB |  | 37.3 |
|  | gene1206 | macB |  | 32.6 |
|  | gene1302 | macB |  | 34 |
|  | gene1383 | macB |  | 39.2 |
|  | gene1407 | macB |  | 26.3 |
|  | gene1471 | macB |  | 34.5 |
|  | gene1585 | oleC | oleC is an ABC transporter isolated from Streptomyces antibioticus and is involved in oleandomycin secretion. | 31.4 |
|  | gene0250 | oleC |  | 29 |
|  | gene1466 | oleC |  | 26.5 |
|  | gene1346 | oleC |  | 32.1 |
|  | gene1193 | oleC |  | 22.5 |
|  | gene0744 | oleC |  | 29.1 |
|  | gene1663 | mefE | mefE is a proton motive efflux pump in Streptococcus pneumoniae that confers resistance to macrolides. It is found on the same operon as mefA and the ABC-efflux pump mel. | 37.9 |
| macrolide antibiotic | gene1188 | mtrA | MtrA is a transcriptional activator of the MtrCDE multidrug efflux pump of Neisseria gonorrhoeae. | 43.3 |
|  | gene1382 | mtrA |  | 45 |
| mupirocin | gene1308 | mupA | An alternative isoleucyl-tRNA synthetase conferring resistance to mupirocin. | 28.7 |
|  | gene0840 | mupB |  | 24.8 |
| nitroimidazole antibiotic | gene0187 | msbA | MsbA is a multidrug resistance transporter homolog from E. coli and belongs to a superfamily of transporters that contain an adenosine triphosphate (ATP) binding cassette (ABC) which is also called a nucleotide-binding domain (NBD). MsbA is a member of the MDR-ABC transporter group by sequence homology. MsbA transports lipid A, a major component of the bacterial outer cell membrane, and is the only bacterial ABC transporter that is essential for cell viability. | 28.3 |
|  | gene0192 | msbA |  | 27.8 |
|  | gene1244 | msbA |  | 29.7 |
|  | gene1806 | msbA |  | 26 |
| nucleoside antibiotic | gene0046 | sta | Streptothricin acetyltransferase gene (STAT gene) that confers streptothricin resistance on Escherichia coli and Bacillus subtilis. | 49.1 |
| peptide antibiotic | gene0080 | ugd | PmrE is required for the synthesis and transfer of 4-amino-4-deoxy-L-arabinose (Ara4N) to Lipid A, which allows gram-negative bacteria to resist the antimicrobial activity of cationic antimicrobial peptides and antibiotics such as polymyxin | 58.2 |
|  | gene1102 | ugd |  | 53.8 |
|  | gene0335 | bcrA | bcrA is an ABC transporter found in Bacillus licheniformis that confers bacitracin resistance | 26.2 |
|  | gene0776 | bcrA |  | 33.5 |
|  | gene0864 | bcrA |  | 35.7 |
|  | gene0983 | bcrA |  | 30.3 |
|  | gene1078 | bcrA |  | 39.5 |
|  | gene1469 | bacA |  | 36.6 |
|  | gene1757 | bcrA |  | 28 |
|  | gene1776 | bcrA |  | 33.8 |
|  | gene1858 | bcrA |  | 37 |
|  | gene1900 | bcrA |  | 53.7 |
|  | gene1209 | mprF | MprF is a integral membrane protein that modifies the negatively-charged phosphatidylglycerol on the membrane surface. This confers resistance to cationic peptides that disrupt the cell membrane, including defensins. | 40 |
| peptide antibiotic; rifamycin antibiotic | gene0331 | rpoB2 | Due to gene duplication, the genomes of Nocardia species include both rifampin-sensitive beta-subunit of RNA polymerase (rpoB) and rifampin-resistant beta-subunit of RNA polymerase (rpoB2) genes, with ~88% similarity between the two gene products. Expression of the rpoB2 variant results in replacement of rifampin sensitivity with rifampin resistance. | 59.4 |
| pleuromutilin antibiotic | gene0694 | TaeA | Pleuromutilin (Tiamulin) ABC efflux pump found in Paenibacillus sp. LC231, a strain of Paenibacillus isolated from Lechuguilla Cave, NM, USA. Confers resistance to pleuromutilin antibiotics. Described by Pawlowski et al. 2016. | 45.1 |
| streptogramin antibiotic | gene0073 | vatB | vatB is a plasmid-mediated acetyltransferase found in Staphylococcus aureus | 32.3 |
|  | gene1105 | vatB |  | 49 |
| sulfonamide antibiotic | gene1799 | sul4 | sul4 is a dihydropteroate synthase gene and mobile sulfonamide resistance gene shown to confer resistance when expressed in E. coli. | 41.2 |
| tetracycline antibiotic | gene0043 | otr(A) | otr(A) is an oxytetracycline resistance ribosomal protection protein found in Streptomyces rimosus | 39.1 |
|  | gene0384 | tetS | Tet(S) is a ribosomal protection protein found in Gram-positive and Gram-negative strains. It is similar to tet(M) and tet(O). | 30.9 |
|  | gene0702 | tetA(46) | tetA(46) is a subunit of tetAB(46), a heterodimeric ABC transporter, that is required for conferring tetracycline resistance in Streptococcus australis isolated from the oral cavity. | 52.2 |
|  | gene0789 | tetA(46) |  | 27.6 |
|  | gene1237 | tetA(46) |  | 35.9 |
|  | gene0105 | tetA(58) | TetA(58) is a Tetracycline efflux pump described in Paenibacillus sp. LC231, a strain of Paenibacillus isolated from Lechuguilla Cave, NM, USA. Described by Pawlowski et al. 2016. | 25.8 |
|  | gene0118 | tetA(58) |  | 32.5 |
|  | gene1885 | tetA(58) |  | 36.5 |
|  | gene1345 | tetA(58) |  | 29.1 |
|  | gene1641 | tetA(58) |  | 31.1 |
|  | gene0850 | tetA(58) |  | 25.2 |
|  | gene0340 | tetA(60) | tetA(60) is a subunit of tetAB(60), an ABC transporter that confers resistance to tetracycline and tigercycline identified by screening a human saliva metagenomic library in Escherichia coli, which is required for resistance. | 25.8 |
|  | gene0972 | tetT | Tet(T) is a ribosomal protection protein of streptococci. It is similar to Tet(Q). | 27.2 |
|  | gene1231 | tet36 | Tet36 is a tetracycline resistance gene found in Bacteroides similar to Tet(Q), and binds to the ribosome to confer antibiotic resistance as a ribosomal protection protein. | 38 |
|  | gene0703 | tetB(46) | tetB(46) is a subunit of tetAB(46), a heterodimeric ABC transporter, that is required for conferring tetracycline resistance in Streptococcus australis isolated from the oral cavity. | 56.6 |
|  | gene1236 | tetB(60) | tetB(60) is a subunit of tetAB(60), an ABC transporter that confers resistance to tetracycline and tigercycline identified by screening a human saliva metagenomic library in Escherichia coli, which is required for resistance. | 38.4 |
|  | gene0190 | tetB(60) |  | 24.4 |
|  | gene0643 | tetB(60) |  | 27.2 |
|  | gene1883 | tetB(P) | TetB(P) is a tetracycline ribosomal protection protein found on the same operon as tetA(P), a tetracycline efflux protein. | 26.3 |
|  | gene0721 | otr(A) | otr(A) is an oxytetracycline resistance ribosomal protection protein found in Streptomyces rimosus | 34.8 |
