# Supplementary figures and images for: Pathogenicity of Streptococcus iniae causing mass mortalities of yellow catfish (Tachysurus fulvidraco) and its induced host immune response
Source: Front Microbiol. 2024 Mar 22;15:1374688. doi: 10.3389/fmicb.2024.1374688 (PMC10995319; doi:10.3389/fmicb.2024.1374688)

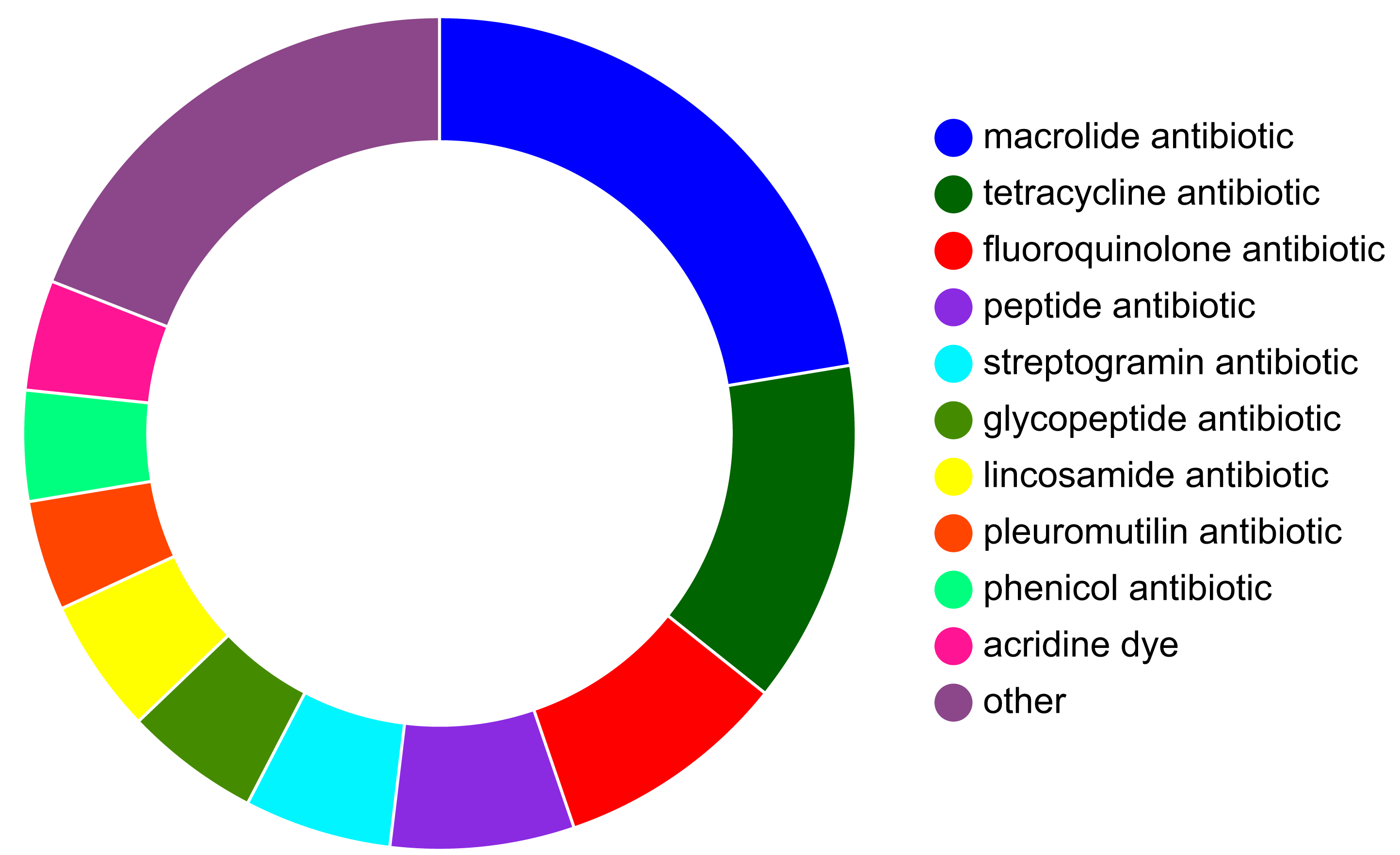

Supplement: Supplementary file 3 [file Image_1.TIF]
